# Age-based partitioning of individual genomic inbreeding levels in Belgian Blue cattle

**DOI:** 10.1186/s12711-017-0370-x

**Published:** 2017-12-22

**Authors:** Marina Solé, Ann-Stephan Gori, Pierre Faux, Amandine Bertrand, Frédéric Farnir, Mathieu Gautier, Tom Druet

**Affiliations:** 1 Unit of Animal Genomics, GIGA-R & Faculty of Veterinary Medicine, University of Liège, B34 (+1) Avenue de l’Hôpital 1, 4000 Liège, Belgium; 2 Awé Coopérative (Association Wallonne de l’Élevage) - Recherche et Développement, Rue des Champs Elysées 4, 5590 Ciney, Belgium; 3 BBASV, FARAH-PAD & Faculty of Veterinary Medicine, University of Liège, Quartier Vallée 2, Avenue de Cureghem, (B43 +3), 4000 Liège, Belgium; 4 INRA, UMR CBGP (Centre de Biologie pour la Gestion des Populations), Campus International de Baillarguet, 34988 Montferrier sur Lez, France; 5 IBD (Institut de Biologie Computationnelle), 34095 Montpellier, France

## Abstract

**Background:**

Inbreeding coefficients can be estimated either from pedigree data or from genomic data, and with genomic data, they are either global or local (when the linkage map is used). Recently, we developed a new hidden Markov model (HMM) that estimates probabilities of homozygosity-by-descent (HBD) at each marker position and automatically partitions autozygosity in multiple age-related classes (based on the length of HBD segments). Our objectives were to: (1) characterize inbreeding with our model in an intensively selected population such as the Belgian Blue Beef (BBB) cattle breed; (2) compare the properties of the model at different marker densities; and (3) compare our model with other methods.

**Results:**

When using 600 K single nucleotide polymorphisms (SNPs), the inbreeding coefficient (probability of sampling an HBD locus in an individual) was on average 0.303 (ranging from 0.258 to 0.375). HBD-classes associated to historical ancestors (with small segments ≤ 200 kb) accounted for 21.6% of the genome length (71.4% of the total length of the genome in HBD segments), whereas classes associated to more recent ancestors accounted for only 22.6% of the total length of the genome in HBD segments. However, these recent classes presented more individual variation than more ancient classes. Although inbreeding coefficients obtained with low SNP densities (7 and 32 K) were much lower (0.060 and 0.093), they were highly correlated with those obtained at higher density (r = 0.934 and 0.975, respectively), indicating that they captured most of the individual variation. At higher SNP density, smaller HBD segments are identified and, thus, more past generations can be explored. We observed very high correlations between our estimates and those based on homozygosity (r = 0.95) or on runs-of-homozygosity (r = 0.95). As expected, pedigree-based estimates were mainly correlated with recent HBD-classes (r = 0.56).

**Conclusions:**

Although we observed high levels of autozygosity associated with small HBD segments in BBB cattle, recent inbreeding accounted for most of the individual variation. Recent autozygosity can be captured efficiently with low-density SNP arrays and relatively simple models (e.g., two HBD classes). The HMM framework provides local HBD probabilities that are still useful at lower SNP densities.

**Electronic supplementary material:**

The online version of this article (10.1186/s12711-017-0370-x) contains supplementary material, which is available to authorized users.

## Background

Two alleles are identical-by-descent (IBD) if they descend from a single allele in an ancestor. This measure is relative and depends on the definition of a reference (or base) population. Indeed, two alleles are declared IBD if the ancestor belongs to the reference population and identical-by-state (IBS) for more remote common ancestors. When two alleles are IBD within an individual, the terms “autozygous” or “homozygous-by-descent” (HBD) are used. The inbreeding coefficient F of an individual is related to these measures and is defined as the probability that two alleles at any locus in this individual are IBD [1]. Inbreeding is associated with negative effects on fitness (e.g., [2–4]) and the occurrence of monogenic disorders increases in populations with higher levels of inbreeding [5]. Thus, the study and management of inbreeding are of high importance in such populations. Belgian Blue Beef cattle (BBB) represent a good example of an intensively selected cattle population. A consequence of the selection process in this breed is the increase in the level of inbreeding, as illustrated by several recent outbreaks of genetic recessive defects [5–11].

There are several methods to estimate the inbreeding coefficient F. In the past, methods were based on the genealogy and estimated the expected inbreeding coefficient (based on the relationship between the two parents). With the development of genetic markers, several approaches allow the estimation of the realized inbreeding coefficient (“observed” in an individual), even in the absence of genealogy. Global approaches, including moments estimators (e.g., [12]), simple homozygosity measures (e.g., [2]) or based on the genomic relationship matrix [13], estimate the total amount of inbreeding in an individual and can work with sparse genetic maps. Methods that are based on runs of homozygosity (ROH) (e.g., [14]) are, most often, empirical rule-based methods, which assume that long stretches of identical alleles are HBD. For such rule-based methods, prior parameters have to be defined, i.e., the minimal number of homozygous markers, the minimal length and the maximum number of allowed heterozygous markers to consider a set of successive markers as HBD, etc. Likelihood-based approaches (e.g., [15, 16]) rely on probabilistic models, which use allele frequencies and genotyping error rates to determine whether ROH are autozygous (i.e., HBD), and derive from earlier works by Broman and Weber [17]. Compared to global estimators, ROH-based methods require denser genetic maps and can provide estimators of local autozygosity. ROH have been used to study inbreeding in diverse species including humans [14, 16, 18], pigs [19], cattle [20, 21] and others, and to study genetic diversity and signatures of selection. In addition, ROH offer the possibility to distinguish between recent and more ancient inbreeding [16, 18, 22]. Indeed, segments that are inherited from recent ancestors are expected to be longer since the recombination process has fewer generations to split the fragment into smaller pieces. Finally, hidden Markov models (HMM) were developed to estimate the HBD probability of segments along chromosomes [23] and make use of all the available information about the sequences of homozygous/heterozygous markers, allele frequencies of markers, the genetic map, and genotyping error rates. These models can handle whole-genome sequence data [24], including low-fold experiments [25]. All these HMM assume that (1) all the autozygosity results from a single event, (2) all the HBD segments trace back to one or several ancestors in a single generation, and (3) they all have the same expected length. However, natural and domesticated populations are complex. They result from a long demographic history with variable effective population size (Ne) and, sometimes, have undergone major demographic events such as bottlenecks.

To relax this strong assumption of the current HMM methods, we recently developed a new HMM with multiple age-based HBD-classes [26] in which the length of the HBD segments from different classes have distinct expected distributions (longer/shorter segments for more recent/ancient common ancestors). The model allows to fit genomic data better and to reveal the “recent” demographic history of populations. The aims of our study were to: (1) characterize inbreeding by using a model describing genomes as a mosaic of non-HBD and HBD segments and partitioning the latter in multiple age-related classes in an intensively selected cattle population such as BBB cattle; (2) investigate the effect of marker density and setting of parameters on the estimates; and (3) compare our estimates with those obtained with other methods (pedigree-based inbreeding coefficients, estimates from the genomic relationship matrix or rule-based ROH estimators).

## Methods

### Data

Single nucleotide polymorphism (SNP) genotypes for the 735,293 SNPs from the Illumina BovineHD BeadChip (HD; Illumina, San Diego, CA) were available for 634 BBB sires. Moreover, whole-genome sequencing (WGS) data were also available for 50 of these sires (the bioinformatic processing of the WGS data is described in [27]). The pedigree including all known ancestors of the 634 bulls contained 7676 individuals. In addition, we extracted from the Widde database (http://widde.toulouse.inra.fr; [28]), Illumina BovineHD genotypes for animals belonging to 10 cattle breeds of European origin (originally provided by the BovineHD genotyping consortium). This set contained samples from 42 Angus, 22 Brown Swiss, 37 Charolais, 21 Guernsey, 35 Hereford, 60 Holstein, 38 Jersey, 50 Limousin, 21 Piedmontese and 21 Romagnola individuals.

All individuals had a call rate higher than 0.90. We selected SNPs that mapped to bovine autosomes (using the UMD3.1 build) and removed from the dataset those that had a call rate lower than 95% and minor allelic frequency lower than 0.01, that significantly deviated from Hardy–Weinberg proportions (p < 0.001) or that presented incompatible genotypes for more than one parent–offspring pair, which resulted in a set of 601,226 SNPs. Furthermore, SNPs located in segments that might be incorrectly mapped to the genome build were removed. Such putative errors were identified based on evidence from linkage information [29], linkage disequilibrium [30] or an excess of breaks in ROH from independent samples [31]. Consequently, an additional 2.7% of the SNPs were filtered out, which resulted in a final BBB dataset of 585,159 SNPs. Removing potential map errors is essential for our applications since these might break long ROH into smaller fragments. For the other breeds, the number of conserved SNPs using the same rules ranged from 524,113 to 622,603 SNPs.

To study the effect of SNP density on the estimation of inbreeding, we used two subsets of the 585,159 SNPs selected for BBB cattle based on their presence on the bovine Illumina BovineSNP50 BeadChip v1 and v2 (32,412 SNPs conserved for this 50 K panel) or on both the 50 K panel and the Illumina BovineLD BeadChip (6844 SNPs conserved for this low-density (LD) panel).

For the sequence data, first we applied stringent filtering rules to select a high-quality subset of SNPs, as described in [31]. Briefly, SNPs, which passed the calibration score and were present in other cattle WGS datasets (1000 bull genomes project [32], Holstein and Jersey individuals from New-Zealand [27] and a Dutch Holstein pedigree of 415 individuals that was used as a reference population for imputation in [33]), were selected, resulting in a set of ancient variants. We conserved only the SNPs that presented correct Mendelian segregation in the WGS Dutch Holstein pedigree (see [33] for more details). Regarding the genotyping data, we also removed variants with a MAF lower than 0.01 and some possibly incorrectly mapped regions (errors in the genome assembly) based on the rules described in [31]. The final WGS dataset contained 5,653,911 bi-allelic SNPs.

### Methods to estimate inbreeding coefficients and HBD probabilities

#### Multiple HBD-classes HMM

Our multiple HBD-classes model [26] is a HMM that describes individual genomes as mosaics of multiple HBD and non-HBD states. Although several non-HBD states can be fitted, here we used only one non-HBD state and *K* − 1 HBD states for a total of *K* states, where *K* is a parameter of the method that can be either predefined or selected by model comparison (see below). Each state *k* has its own rate parameter *R*_*k*_ that defines the distribution of the lengths of the segments originating from that class: the lengths in Morgans are distributed exponentially with rate *R*_*k*_. The rate corresponds approximately to the size of the inbreeding loop measured in generations and is closely related to age in generations of the common ancestors. *R*_*k*_ is approximately twice the number of generations to the common ancestor. Each state has also its own mixing proportion, which is equal to the frequency of segments originating from that class. Such a model with multiple-HBD classes will be referred to as a KR model, with *K* being equal to the number of distinct rates fitted, *K* − 1 for HBD states and 1 for the non-HBD state. In the case where a single HBD class and a single non-HBD class are fitted, we use a common rate for both (1R model) since such a model has better properties [26]. Emission probabilities of the HMM correspond to the probabilities of observing a particular genotype conditionally on the underlying state (HBD or non-HBD). For non-HBD classes, these probabilities correspond to Hardy–Weinberg proportions [26] and for HBD classes, homozygotes *AA* are observed with a probability *f*_*A*_ (1 − $\upvarepsilon$) and heterozygotes with a probability $\upvarepsilon$, where *f*_*A*_ is the frequency of allele *A* and $\upvarepsilon$ is an error term corresponding to the probability of observing a heterozygous genotype in a HBD segment [26]. With WGS data, these probabilities are integrated over the different genotype probabilities obtained from the VCF file [26]. For each HBD class, the genome-wide HBD probability is estimated as the probability of belonging to that class averaged over the whole genome, whereas the local HBD probability is defined as the probability of belonging to that class at a specific genomic location (see [26] for more details). The genome-wide HBD probabilities correspond to the percentage of the genome that is associated with a specific HBD class, e.g., the proportion of the genome that is located within HBD segments of a certain length. To estimate the inbreeding coefficient, first the base population must be defined, which is done by deciding which classes are considered as truly autozygous. For instance, we might consider that ancestors associated with classes with a *R*_*k*_ rate higher than a selected threshold *T* (i.e., *R*_*k*_ ≥ *T*) are unrelated. Then, the corresponding inbreeding coefficient *F*_*G*-*T*_ is estimated as the probability to belong to any of the HBD classes with a *R*_*k*_ ≤ *T* averaged over the whole genome (e.g., the inbreeding coefficient is defined as the probability of sampling an HBD locus given a reference population). Since *R*_*k*_ rates of HBD classes are approximately equal to twice the number of generations to the common ancestor, including HBD classes with a *R*_*k*_ ≤ *T* amounts to setting the base population to approximately 0.5 * *T* generations ago. In the remainder of the manuscript, inbreeding coefficients or HBD probabilities reported without specifying a base population or *R*_*k*_, are obtained by including all HBD classes (e.g., using the most remote base population). In that case, the age of the base population or the smallest HBD segments captured are a function of the SNP density used. All the HBD probabilities are estimated with the forward–backward algorithm [34].

As an alternative to the KR model, we can use a set of pre-defined *R*_*k*_ rates and estimate only the mixing proportions (MixKR model). This set of *R*_*k*_ rates should be selected to cover a wide range of past generations. In our analyses, we used 13 HBD states with respective *R*_*k*_ rates equal to [2^1^, 2^2^, 2^3^, …, 2^13^] and one non-HBD class with a rate of 2^13^. These values were chosen to have a constant and limited degree of overlap between the exponential distributions that specify the HBD lengths for each successive class. The upper rate is determined by the SNP density that defines the size of the smallest HBD segments that we can capture. Such models proved efficient to estimate the genome-wide (global) and local autozygosity levels and to obtain information on recent demographic history [26]. In addition, inbreeding coefficients are then estimated with respect to the same reference population and HBD classes are defined over identical periods in the past, allowing better comparisons between individuals.

With all the models, the parameters (mixing proportions for all models and *R*_*k*_ rates for KR models only) were estimated with 1000 iterations of the expectation-maximization algorithm with constraints to force *R*_*k*_ to be between 1 and 8192. The number of classes *K* is fixed for each run but the optimal value can be determined by comparing models with the Bayesian information criterion (BIC). All analyses were performed with the ZooRoH software (https://github.com/tdruet/ZooRoH).

#### Additional inbreeding coefficient estimators

The inbreeding coefficient based on pedigree data (F_PED_) was computed with the method of Sargolzaei et al. [35]. We used several measures to estimate genomic inbreeding coefficients. The first measure uses the diagonal elements of the genomic relationship matrix (GRM) computed with the BLUPF90 package [36] without any pedigree information ($\upalpha$ set to 1.0) and is based on the variance of the additive genetic values (F_GRM_; [13, 37]). The second, which was proposed and recommended by Yang et al. [38] for its smaller sampling variance, is based on the correlation between uniting gametes (F_UNI_) and was estimated using the GCTA software [39]. The third more simple measure is defined as the homozygosity (F_HOM_) or the proportion of homozygous SNPs (e.g., [2]), which is closely related to the excess homozygosity estimator (F_ExHOM_) implemented in plink [40]. For F_GRM_, F_UNI_ and F_ExHOM_, we estimated allele frequencies based on the 31 bulls born before 1985. Finally, the fourth estimator measures the proportion of the genome covered by ROH (F_ROH_), which contained at least 15 SNPs and were identified using plink [40] with 50-SNP windows (no heterozygous genotypes were accepted and up to five missing genotypes were possible). These parameters were selected based on published studies in cattle (e.g., [20, 22, 41]). The minimal SNP density, length of ROH and maximal SNP spacing were optimized for each panel as follows by order of increasing density: at least one SNP per 500, 100 and 10 kb, the length of ROH had to be at least 5 Mb, 1 Mb and 100 kb long and the maximum distance between two consecutive SNPs had to be 1 Mb, 500 kb and 200 kb.

## Results

### Estimation and age-based partitioning of individual genomic inbreeding levels in the Belgian Blue Beef cattle population

**Fig. 1:**
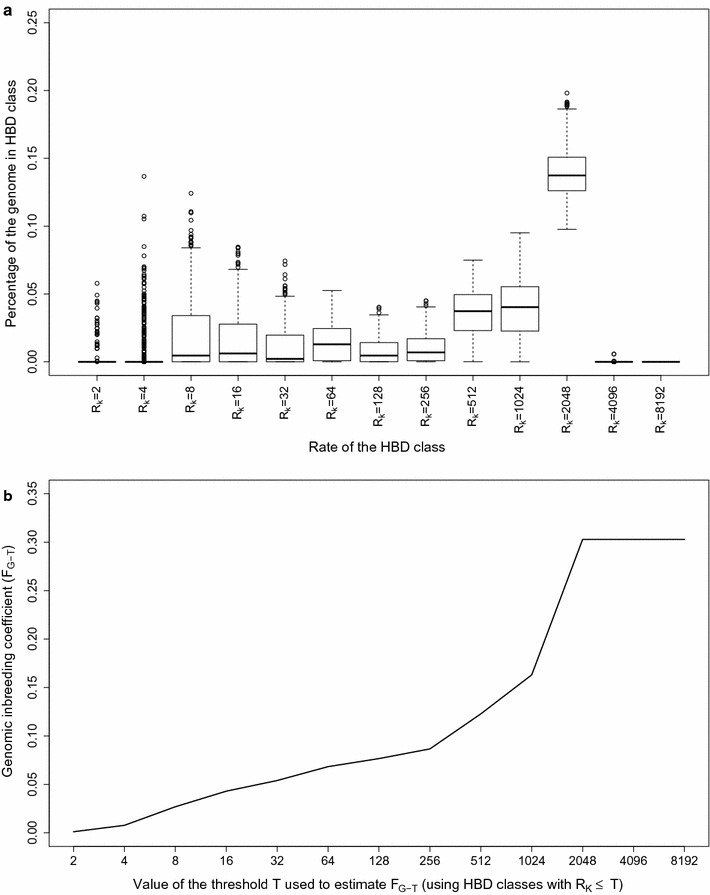
Partitioning of genome-wide autozygosity for the 634 Belgian Blue sires using the BovineHD SNP panel. **a** Boxplot of percentages of individual genomes associated with 13 HBD-classes with pre-defined *R*_*k*_ rates (Mix14R model). The percentages correspond to individual genome-wide probabilities of belonging to each of the HBD-classes. **b** Genomic inbreeding coefficients estimated with respect to different base populations (*F*_*G*-*T*_) obtained by selecting different thresholds *T* that determine which HBD-classes are considered in the estimation of *F*_*G*-*T*_ (e.g., setting the base population approximately 0.5 * *T* generations in the past). The corresponding inbreeding coefficients *F*_*G*-*T*_ are estimated as the probability of belonging to any of the HBD classes with a *R*_*k*_ ≤ *T* averaged over the whole genome

We started by using a Mix14R model (with *R*_*k*_ ranging from 2 to 8192) to estimate the proportion of the genome belonging to different HBD classes for the 634 BBB sires (Fig. 1a), which allows the estimation of the inbreeding coefficient with respect to different base populations as explained in Methods (Fig. 1b). When considering all HBD classes, the fraction of the genome that is HBD (corresponding to the inbreeding coefficient estimated with the most remote base population) was equal to 0.303 on average (ranging from 0.258 to 0.375), with a major contribution from HBD-classes with high *R*_*k*_ rates (*R*_*k*_ > 256) that account for 71.4% of the total HBD proportion on average. These small ROH reflect the history of the population (background inbreeding and linkage disequilibrium associated with past effective population size (N_e_)) better than individual variation. Classes associated with smaller *R*_*k*_ rates (i.e., with longer HBD segments) accounted for a smaller proportion of the total HBD proportion (the average inbreeding coefficient was equal to 0.054 and 0.087 when including HBD-classes with *R*_*k*_ ≤ 32 and *R*_*k*_ ≤ 256, respectively, and setting the base population approximately 16 or 128 generations ago) but presented more variation among individuals. For instance, the inbreeding coefficient associated with common ancestors tracing back up to approximately four generations ago (corresponding to HBD-classes with *R*_*k*_ ≤ 8) ranged from 0.000 to 0.137. For bulls born from 1980 to 2010, the percentage of the genome in HBD segments increased by 3.3% (+ 0.11% per year), i.e., approximately from 28 to 31% (see Additional file 1: Fig. S1a). However, the trend for more recent HBD classes (*R*_*k*_ ≤ 32) was more pronounced (see Additional file 1: Fig. S1b), i.e., from almost 0 to 6% (+ 0.20% per year) and corresponded more closely to the trend observed with pedigree-based inbreeding coefficients (see Additional file 1: Fig. S1c). Bulls born before 1980 presented little evidence of recent autozygosity compared to modern bulls.

**Fig. 2:**
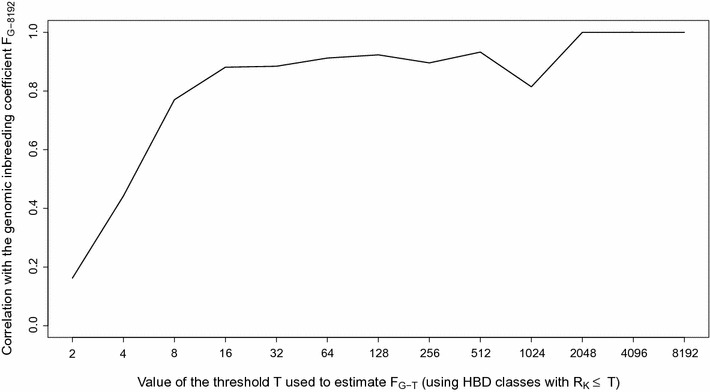
Correlations between genomic inbreeding coefficients estimated with respect to different base populations (*F*_*G*-*T*_) and the inbreeding coefficient estimated with the most remote base population F_G-8192_ (including all HBD classes). Different base populations are obtained by selecting different thresholds *T* that determine which HBD-classes are considered in the estimation of *F*_*G*-*T*_ (e.g., setting the base population approximately 0.5 * *T* generations in the past). The corresponding inbreeding coefficients *F*_*G*-*T*_ are estimated as the probability of belonging to any of the HBD classes with a *R*_*k*_ ≤ *T* averaged over the whole genome. Estimation of inbreeding coefficients was performed with the Mix14R model (13 HBD-classes model with pre-defined *R*_*k*_ rates) for 634 Belgian Blue sires and using the BovineHD SNP panel

To assess the contribution of each HBD class to the percentage of the genome in HBD segments and to its variation in BBB cattle, we divided the total fraction of the genome in HBD classes [0.303 on average; standard deviation (SD) = 0.071] in four main classes (very recent HBD classes with *R*_*k*_ = 2 to 8, recent HBD classes with *R*_*k*_ = 16 to 64, ancient HBD classes with *R*_*k*_ = 128 to 512, and very ancient HBD classes with *R*_*k*_ = 1024 to 8192), with each group having three HBD classes except the last one with four HBD classes. The average fraction of the genome associated with each of these main classes (ordered from recent to ancient) was equal to 0.027 (SD = 0.029), 0.041 (SD = 0.019), 0.054 (SD = 0.013) and 0.180 (SD = 0.011). Note that high proportions of very recent HBD segments are mechanically associated with lower proportions of very ancient HBD segments (r = − 0.407) because recent HBD segments mask more ancient HBD segments. Although the percentage of the genome in HBD classes associated with recent common ancestors represents only 22.6% of the total autozygosity, it displays more individual variation than that in more ancient classes (more than 50% of the total variance is associated with very recent HBD classes). By fitting a linear model, we estimated that very recent HBD classes account for 59% of the total autozygosity variation and that adding recent HBD classes to the model increases this value to 83%. Similarly, the correlations between inbreeding coefficients measured with respect to different base populations (e.g., including different HBD classes in the computation) with the inbreeding coefficients estimated using all HBD classes increased abruptly from 0.16 for estimates based on the first class (*R*_*k*_ = 2) to 0.77 for inbreeding coefficients estimated including HBD classes with a *R*_*k*_ ≤ 8 and to 0.90 with a *R*_*k*_ ≤ 16, and then improved only marginally by adding more HBD-classes (Fig. 2). The decrease in correlation observed at *R*_*k*_ = 1024 results from the fact that ancient autozygosity is concentrated at *R*_*k*_ = 1024 for some individuals and at *R*_*k*_ = 2048 for others.

### Comparison of the results for BBB cattle with those of other breeds

**Fig. 3:**
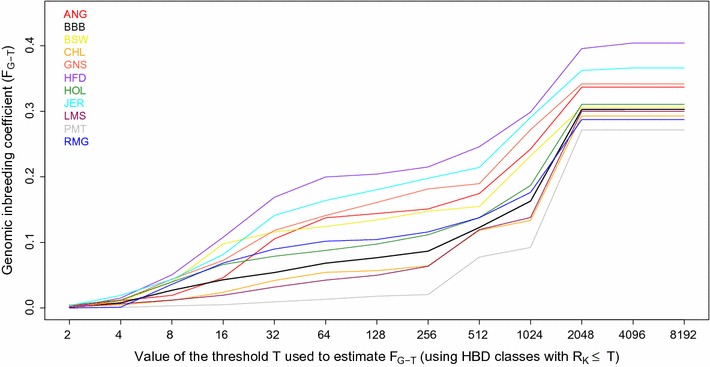
Estimation of inbreeding coefficients with respect to different base populations (the threshold *T* determines which HBD classes are included in the estimation of *F*_*G*-*T*_) with a Mix14R model in 11 cattle breeds of European origin using the BovineHD SNP panel. *ANG* Angus, *BBB* Belgian Blue Beef cattle, *BSW* Brown Swiss, *CHL* Charolais, *GNS* Guernsey, *HFD* Hereford, *HOL* Holstein, *JER* Jersey, *LMS* Limousin, *PMT* Piedmontese, *RMG* Romagnola

To determine whether comparable levels and patterns of autozygosity are also observed in other breeds of European origin, we applied the same model to 10 breeds genotyped with the same array (Fig. 3). In most of these breeds, inbreeding coefficients estimated with respect to different base populations increased moderately up to F_G-256_ (e.g., HBD-class with *R*_*k*_ ≤ 256 included in the estimation) and more strongly with older base populations (F_G-512_ to F_G-2048_), which include many more generations of ancestors. Large differences in inbreeding coefficients were observed with relatively recent base populations (F_G-64_, approximately 32 generations ago), ranging from 0.013 and 0.042 in Piedmontese and Limousin to 0.164 and 0.200 in Jersey and Hereford cattle. Some Hereford individuals presented extreme inbreeding coefficients estimated with recent base populations (see Additional file 2), i.e., up to 40% for F_G-8_ (e.g., approximately four generations back). Part of the Hereford individuals from this dataset come from the Hereford Line 1, an inbred line, which indicates that our model captures extreme events correctly but also that genotyped individuals included in this study are not necessarily representative of the breed.

### Estimation of inbreeding coefficients and HBD probabilities with different SNP densities

**Fig. 4:**
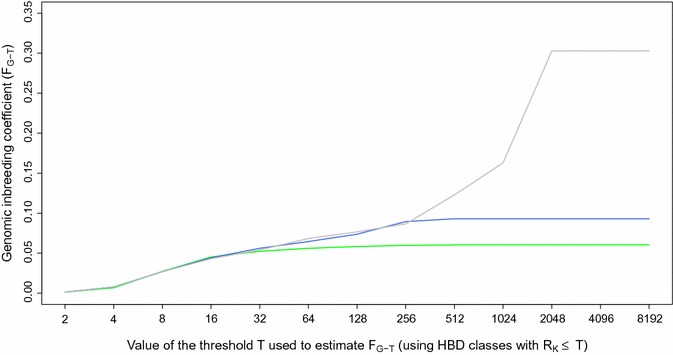
Comparison of inbreeding coefficients estimated with different SNP densities (LD panel in green, 50 K panel in blue and BovineHD panel in grey) and for different base populations (the threshold *T* determines which HBD classes are included in the estimation of *F*_*G*-*T*_). Estimation of inbreeding coefficients was performed with the Mix14R model for 634 Belgian Blue sires

We fitted a Mix14R model using different SNP densities, i.e., from LD (6844 SNPs) to HD (601,226 SNPs) on the 634 BBB dataset and even to WGS (5,653,911 SNPs) for the 50 whole-genome sequenced individuals. Average estimated inbreeding coefficients measured with respect to different base populations (Fig. 4) and Additional file 3: Fig. S2 were similar across SNP panels for the most recent base populations (F_G-32_). For more ancient base populations, less autozygosity was captured with the LD panel with marked differences for ancient HBD classes that were captured only with HD or WGS panels. A similar trend was observed with the 50 K panel but average inbreeding coefficients were similar to those from the HD panel up to F_G-256_ (approximately 128 generations back). The average inbreeding coefficients estimated by using the most remote base population and the LD, 50 K and HD panels were equal to 0.060, 0.093 and 0.303, respectively (when estimated on the 50 sequenced individuals only, these values were equal to 0.047, 0.101 and 0.309, respectively, and to 0.359 with the WGS panel). The base population is then a function of the smallest HBD segments that can be captured by the panel used. The correlations between these inbreeding coefficients estimated with different panels were high, i.e., 0.934 (LD-HD), 0.944 (LD-50 K) and 0.975 (50 K-HD). In spite of the much lower inbreeding coefficients obtained with the 50 K panel, it captures essentially all the individual variation obtained with a HD panel, in agreement with the earlier observation that most of the variation was associated with recent HBD classes.

**Fig. 5:**
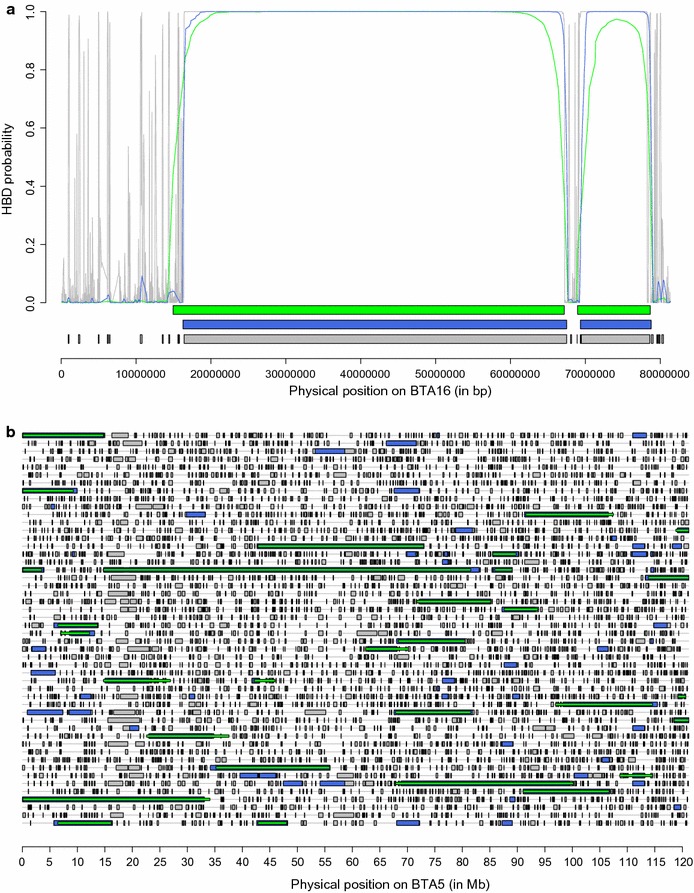
Illustrations of the identification of HBD segments using different SNP panels. **a** Example of estimated HBD probabilities for one individual on *Bos taurus* autosome (BTA) 16 using different SNP densities (LD panel in green, 50 K panel in blue and BovineHD panel in grey). The horizontal lines below the curves represent HBD segments as identified by the Viterbi algorithm with the three panels. An extremely long HBD segment (~ 50 Mb) is represented (there are only 69 such HBD segments identified in the entire data set), suggesting recent inbreeding. This bull is one of the 29 individuals carrying such long HBD segments and has a pedigree inbreeding coefficient of 0.048. **b** Comparisons of HBD segments identified for 50 individuals on BTA5 using different panels (each line represents one individual). Segments identified with the HD, 50 K and LD panels are represented in grey, blue and green, respectively (with lower density results masking results obtained at higher density). The shortest HBD segments are identified with the HD panel (indicated in grey) whereas those of intermediate size are also captured with the 50 K panel (and still missed with the LD panel) and indicated in blue. For a few HBD segments, the use of the LD panel results in longer segments

**Fig. 6:**
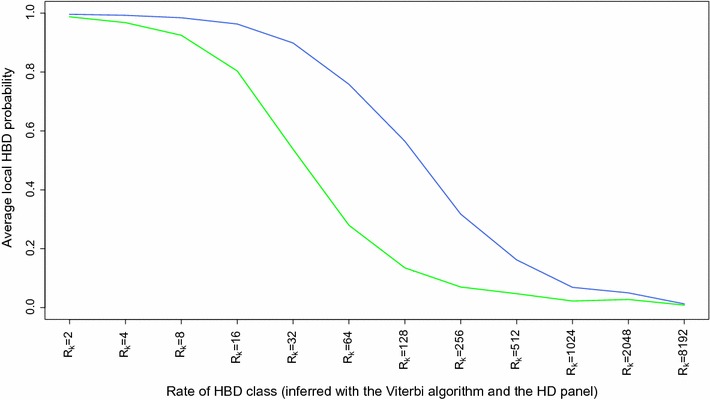
Average HBD probabilities estimated for HBD segments associated with different age-based classes. HBD probabilities were estimated with the LD (green) or 50 K (blue) panels whereas the age-based classes were determined by using the Viterbi algorithm and the HD panel (a 20-fold SNP density increase). The average HBD probabilities indicate whether segments from different classes are captured using lower density panels

We then used the Viterbi algorithm to identify HBD segments with different SNP panels (Table 1). The Viterbi algorithm classifies each SNP position as HBD or non-HBD whereas the forward-backward algorithm provides the local HBD probability. As expected, more and shorter HBD segments are captured with higher density panels. With the HD panel, a limited proportion of extremely small (a few kb) segments were captured. The length of the majority of the segments ranged from 10 to 500 kb, with more than half being shorter than 100 kb, but such segments do not necessarily have the largest contribution to the total percentage of the genome in HBD classes since classes with fewer but longer segments can account for a large proportion of autozygosity. We also observed extremely long HBD segments (> 50 Mb), which confirmed the presence of recent autozygosity (the longest HBD segment was more than 90 Mb long). On average, each of the 634 bulls had 4.25 HBD segments that were longer than 10 Mb and associated to a common ancestor that was present approximately five generations back. The number of such HBD segments ranged from 0 to 14 per individual. Sixty-one bulls had even one or more (up to three) HBD segments longer than 50 Mb. With the 50 K and LD panels, more than 99% of the identified segments were longer than 100 and 500 kb, respectively (with a peak in the classes from 1 to 5 Mb and from 5 to 10 Mb, respectively), and only a fraction of the segments were captured compared to when the HD panel was used. In particular, the vast majority of the HBD segments shorter than 1 Mb were not identified. At lower SNP densities, the smallest segments are simply not captured because they do not contain any SNP or too few. Segments of intermediate size might not reach high HBD probabilities due to a smaller number of SNPs in the segment. Conversely, the length of some HBD segments can be overestimated when using the LD panel, for instance when there are not enough SNPs to identify small non-HBD segments that flank HBD segments. Figure 5a illustrates the identification of HBD segments for one chromosome. It shows that (1) more segments were identified at higher density, (2) HBD probabilities were higher with denser maps, (3) the Viterbi algorithm declared some SNP positions as HBD although they had only moderate HBD probabilities, and (4) the boundaries of HBD segments varied with the panel density. Similarly, Fig. 5b represents HBD segments that were identified on *Bos taurus* chromosome (BTA) 5 for 50 individuals with the Viterbi algorithm with different SNP densities. The results are in agreement with those reported in Table 1. Larger proportions of the genome were declared HBD with the HD panel and small HBD segments accounted for most of the difference with results from lower density panels. Still, we observed that some HBD segments of a few Mb long were not identified at lower SNP density (and even more so with the LD panel). As for Fig. 5a, the length of some HBD segments is overestimated when the LD panel was used. We also compared the local HBD probabilities estimated by using either the LD or the 50 K panel with the local HBD classes inferred by using the HD panel and the Viterbi algorithm (Fig. 6). HBD probabilities were high for recent HBD classes and dropped for more remote common ancestors. As expected, the LD panel was efficient for only the most recent common ancestors (the HBD probability was 0.90 or higher when *R*_*k*_ < 16 and ~ 0.50 for *R*_*k*_ = 32) whereas the 50 K panel allowed the capture of more ancient autozygosity (the HBD probability was 0.90 or higher when *R*_*k*_ < 64 and ~ 0.50 for *R*_*k*_ = 128). More results regarding the age (or length) of HBD segments that can be captured with different SNP densities are described in Druet and Gautier [26].

**Table 1:** Distribution of the length of HBD segments identified with a model with 13 HBD-classes with pre-defined *R*_*k*_ rates for different SNP densities

HBD segment length category	Panel density		
	LD panel	50 K panel	BovineHD panel
≤1 kb	0	0	17
1–5 kb	0	0	1828
5–10 kb	0	0	16296
10–50 kb	1	10	570179
50–100 kb	3	40	614787
100–500 kb	48	1346	793645
0.5–1 Mb	146	2500	53984
1–5 Mb	1172	11658	25839
5–10 Mb	1728	3201	3189
10–50 Mb	2638	2643	2627
50–100 Mb	74	71	69

### Comparison of models

#### Models that estimate *R*_*k*_ rates of HBD-classes (KR models)

**Fig. 7:**
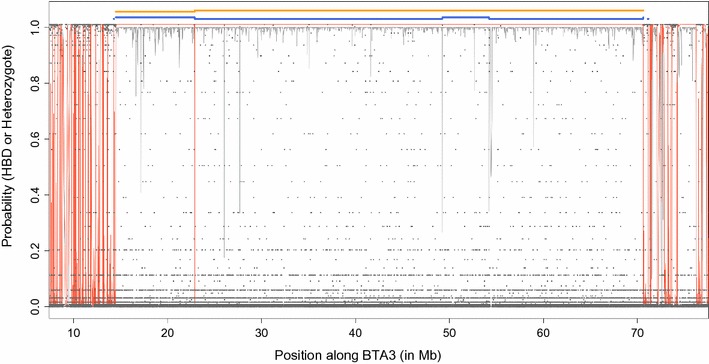
Comparison of the length of HBD segments identified with WGS data and with the 1R or the Mix14R models on BTA3. The grey and red lines represent the HBD probabilities estimated with the 1R and Mix14R models, respectively; the dark grey dots represent the probability of heterozygous genotypes (obtained from the VCF); the blue and yellow segments represent HBD segments identified with the Viterbi algorithm with the 1R and the Mix14R model, respectively

For the different SNP densities tested and for each individual, we used the BIC (see [26]) to select the KR model with the best statistical support (i.e., with the optimal number of classes *K*, with *K* − 1 HBD classes and one non-HBD class) after estimating the rate(s) *R*_*k*_ for each individual with each tested model. For each SNP panel, Table 2 shows the number of times a model was selected as the best one for the individual analyzed. As SNP density increases, more past generations can be explored and the optimal *K* increases accordingly. In most cases, models with one HBD class are preferred for the LD panel, models with two HBD classes for the 50 K panel, models with three HBD classes for the HD and WGS panels (although the model with four HBD classes is also often selected for the latter, i.e., for 23 of 50 individuals). With these optimal models, the first HBD-class captures the most recent autozygosity (*R*_*k*_ from 15 to 20), the second HBD-class captures autozygosity that is associated to common ancestors from a few hundred generations back and later classes are associated with higher *R*_*k*_ (> 1000) (Table 2). Correlations of inbreeding coefficients estimated with these selected KR models with those obtained with the complete Mix14R model (ranging from 0.981 to 1.000) and comparison of the average estimated inbreeding coefficients indicate that with these reduced KR models, we can effectively capture the genome-wide autozygosity. With 1R models and low or moderate SNP densities, we observed a slight underestimation of the inbreeding coefficients compared to the Mix14R model and slightly lower correlations (still above 0.98). The *R*_*k*_ rates estimated for each individual with these panels have a lower median value (respectively 15 and 41 with the LD and 50 K panels) than the *R*_*k*_ rates estimated with higher density panels (median *R*_*k*_ > 1000) for which the contribution of smaller ROH is much larger. As a result, some small fragments were not captured by the model at lower density whereas at higher density, inbreeding coefficients are almost identical to estimates obtained with the Mix14R model. Models containing two or more HBD classes captured the same amount of autozygosity as the Mix14R model, irrespective of SNP density. Although the inbreeding coefficient is correctly estimated with a 1R model (one HBD and non-HBD class with the same rate) with WGS data, the HBD segments identified tend to be smaller since the estimated *R*_*k*_ rates are higher (i.e., smaller expected lengths of fragments) as shown in Additional file 4: Fig. S3. Indeed, the 1R model results in more 10 to 100 kb long segments than the Mix14R model, but fewer segments longer than 100 kb. Thus, with a 1R model, long HBD segments might be cut into smaller fragments in the presence of heterozygous SNPs (possibly sequencing errors) whereas with models including HBD class(es) associated with recent common ancestors (with small *R*_*k*_ rates), these HBD segments are identified as one long and recent fragment (because the penalty to end and start a new segment is higher). Figure 7 illustrates this with an example. Indeed, we observed a long segment with high HBD probabilities although there are multiple positions where the probability of the heterozygous genotype is non-null (but this is limited compared to flanking regions). With the Mix14R model, this is considered as a long segment and the local HBD probability remains higher than 0.99 for the entire region (except for a region with five consecutive heterozygous SNPs). With the 1R model, the HBD probabilities drop repeatedly due to these possibly heterozygous SNPs and the longest HBD segment is cut into several smaller fragments (based on the results from the Viterbi algorithm). Note that with the HD panel, this individual is homozygous for all 13,009 SNPs that are included in this 56.1-Mb long segment. As in Fig. 5, we note that the Viterbi algorithm classifies some positions with a low estimated HBD probability as HBD.

**Table 2:** Comparison of models used to estimate genomic inbreeding coefficients with different numbers of HBD classes (from 1 to 4)

Panel density	N^a^	Number of fitted HBD classes	Mean F_G_	Correlation with F_G-8192_^b^	Median of estimated *R*_*k*_ rates per HBD class			
					1st HBD class	2nd HBD class	3rd HBD class	4th HBD class
LD	634	1	0.058	0.982	15			
LD	0	2	0.061	0.998	11	106		
LD	0	3	0.061	0.999	11	104	162	
LD	0	4	0.061	0.999	10	42	150	175
50 K	289	1	0.083	0.983	41			
50 K	345	2	0.094	0.999	15	198		
50 K	0	3	0.094	1.000	14	173	238	
50 K	0	4	0.094	1.000	11	64	240	243
HD	0	1	0.297	0.999	1214			
HD	0	2	0.302	1.000	60	1679		
HD	629	3	0.303	1.000	22	392	1887	
HD	5	4	0.303	1.000	19	342	1823	1914
WGS	0	1	0.354	1.000	3740			
WGS	0	2	0.359	1.000	577	8158		
WGS	27	3	0.359	1.000	55	1009	8192	
WGS	23	4	0.359	1.000	21	206	1104	8192

The *R*_*k*_ rates of each HBD class were estimated for each individual and for each SNP density (LD, 50 K, HD panels or whole-genome sequence data). The table reports which models are selected as best based on the BIC criterion, the average F_G_ and its correlation with a reference F_G_ obtained with a model using 13 HBD classes

^a^N = number of individuals with the corresponding model selected as best based on the BIC

^b^The reference inbreeding coefficient F_G-8192_ is obtained with a Mix14R model and the same SNP density

#### Models with pre-defined *R*_*k*_ rates of HBD classes (MixKR models)

Compared to the KR models, MixKR models present the advantage of using the same HBD classes for all individuals (*R*_*k*_ rates of HBD classes are not individually estimated but pre-defined by the user) and make comparisons between individuals easier (for instance, comparing two individuals with a single HBD class but with *R*_*k*_ = 8 for the first and *R*_*k*_ = 96 for the second would not be easy – the estimated *R*_*k*_ range from 4 to 1000). Several of these MixKR models (with *K* = 2, 3 and 4) were tested with the LD panel (Table 3) to assess whether reduced models with pre-defined rates of HBD classes are efficient. To select these pre-defined rates, either we used medians of estimated rates obtained from models with the same number of classes (see previous section) or we selected a few classes from the MixKR model in order to cover the range of estimated values (e.g., one class for recent HBD segments and one for ancient HBD segments). In agreement with previous observations on KR models, comparisons of estimated inbreeding coefficients with those obtained with the Mix14R model indicate that models with a single HBD class slightly underestimate the inbreeding coefficients and result in lower correlations (> 0.96) than models with two or more HBD classes (> 0.99). Presence of multiple HBD-classes (> 2) allows better assessment of the contributions from different past generations (e.g., *R*_*k*_ = 8 vs 64) but does not provide better estimates of the genome-wide inbreeding coefficient.

**Table 3:** Estimation of genomic inbreeding coefficients with models using different numbers of HBD classes (from 1 to 4) with pre-defined *R*_*k*_ rates that correspond to the expected length in Morgans of HBD segments and with the LD panel

Number of fitted HBD classes	Mean F_G_	Correlation with reference F_G_^a^	Predefined *R*_*k*_ rates used for each HBD class		
			1st HBD class	2nd HBD class	3rd HBD class
1	0.058	0.967	20		
1	0.056	0.963	16		
2	0.060	0.996	10	100	
2	0.061	0.994	16	256	
3	0.061	0.996	8	64	256

^a^The reference inbreeding coefficient F_G_ is obtained with a Mix14R model and the same SNP density

### Comparison to other inbreeding coefficient estimators

Means and ranges of inbreeding coefficients estimated with different methods and the HD panel are in Table 4 and their correlations are in Table 5, and in Additional file 5: Tables S1 and S2 for other panels. Similar to our model, models based on observed homozygosity and ROH resulted in high inbreeding coefficients (respectively, 0.644 and 0.151 on average) whereas other genomic estimators resulted in inbreeding coefficients centered around 0 and including negative values. It should be noted that higher values are obtained on average (0.268) when using less stringent rules to identify ROH (e.g., windows of 20 SNPs and at least 10 SNPs per ROH). We observed very high correlations between HMM-based estimates and both measures based on homozygosity (r = 0.95 with F_HOM_ and F_ExHOM_, these two measures presenting a correlation of 1 and being essentially the same) or on ROH (r = 0.95 with F_ROH_), which suggest that with large numbers of SNPs, simple heuristics (ignoring allele frequencies, SNP spacing, etc.) are efficient (F_HOM_ and F_ROH_ being highly correlated, r = 0.97). The correlation between F_HOM_ estimated with LD and HD panels is equal to 0.890, which is slightly lower than the correlation between estimates obtained with the HMM for these two panels (r = 0.934), which indicates that global estimators still work properly with 6844 SNPs in this population. Rule-based ROH methods are less efficient at lower SNP densities since they capture only the longest fragments (5 Mb or more and 20 Mb on average) with the parameters used in the current study (the default windows size in plink). In fact, ROH-based estimators are rarely used with the LD panel in cattle although more HBD segments might be identified with less stringent rules, at the expense of an increased rate of false positives. At low SNP density, the HMM framework still provides correct global and local HBD probabilities although HBD segments are not identified without ambiguity [26].

**Table 4:** Summary statistics for the inbreeding coefficients estimated for the 634 Belgian Blue sires with different methods and using the HD panel

Estimators	mean F	min F	max F
F_G_	0.303	0.258	0.375
F_HOM_	0.644	0.621	0.683
F_ExHOM_	− 0.001	− 0.066	0.111
F_ROH_	0.151	0.098	0.237
F_GRM1_	0.031	− 0.150	0.150
F_GRM2_	0.059	− 0.194	0.245
F_UNI_	0.017	− 0.092	0.139
F_PED_*	0.042	0.004	0.091

*F*_*G*_, inbreeding coefficient estimated as the probability of belonging to any of the HBD classes averaged over the whole genome; *F*_*HOM*_, inbreeding coefficient based on the proportion of homozygous SNPs; *F*_*ExHOM*_, excess homozygosity estimator; *F*_*ROH*_, inbreeding coefficient estimated as the proportion of the genome captured by ROH; *F*_*GRM1*_, inbreeding coefficient based on the diagonal elements of the genomic relationship matrix (dividing all SNP contributions by the same denominator); *F*_*GRM2*_, inbreeding coefficient based on the diagonal elements of the genomic relationship matrix (dividing each SNP contribution by its own weight 2*f*_*i*_ (1 − *f*_*i*_), *f*_*i*_ being the frequency of allele *i*); *F*_*UNI*_, inbreeding coefficient based on the correlation between uniting gametes; *F*_*PED*_, inbreeding coefficient estimated from pedigree data

*Estimated on the 313 bulls born after 1999

**Table 5:** Correlations between inbreeding coefficients estimated for the 634 Belgian Blue sires with different methods and using the HD panel

	F_HOM_	F_ExHOM_	F_ROH_	F_GRM1_	F_GRM2_	F_UNI_	F_PED_
F_G_	0.948	0.945	0.945	0.730	0.481	0.905	0.463
F_HOM_		0.999	0.974	0.627	0.343	0.873	0.546
F_ExHOM_			0.974	0.633	0.351	0.878	0.547
F_ROH_				0.610	0.328	0.853	0.551
F_GRM1_					0.938	0.917	0.286
F_GRM2_						0.748	0.091
F_UNI_							0.454

*F*_*G*_, inbreeding coefficient estimated as the probability of belonging to any of the HBD classes averaged over the whole genome; *F*_*HOM*_, inbreeding coefficient based on the proportion of homozygous SNPs; *F*_*ExHOM*_, excess homozygosity estimator; *F*_*ROH*_, inbreeding coefficient estimated as the proportion of the genome captured by ROH; *F*_*GRM1*_, inbreeding coefficient based on the diagonal elements of the genomic relationship matrix (dividing all SNP contributions by the same denominator); *F*_*GRM2*_, inbreeding coefficient based on the diagonal elements of the genomic relationship matrix (dividing each SNP contribution by its own weight 2*f*_*i*_ (1 − *f*_*i*_), *f*_*i*_ being the frequency of allele *i*); *F*_*UNI*_, inbreeding coefficient based on the correlation between uniting gametes; *F*_*PED*_, inbreeding coefficient estimated from pedigree data

Correlations of estimates from the traditional GRM with our estimates are moderately high (r = 0.73) and lower with homozygosity estimators (r = 0.63) and ROH-based estimators (0.61). The F_GRM_ was computed with the formula proposed by [13], which divides all SNP contributions by the same weight. When estimated with the alternative formula, which divides each SNP contribution by its own weight 2*f*_*i*_ (*1* − *f*_*i*_) (*f*_*i*_ being the frequency of SNP *i*) as in Amin et al. [42], correlations were lower (i.e., 0.48 with F_G_, 0.34 with F_HOM_ and 0.33 with F_ROH_). The estimator based on the unified correlations between gametes proposed by Yang et al. [38] presented relatively high correlations with both F_G_ and F_GRM_ (respectively, 0.90 and 0.92) and slightly lower correlations with the other estimators (r = 0.87 and 0.85 with F_HOM_ and F_ROH_, respectively).

**Fig. 8:**
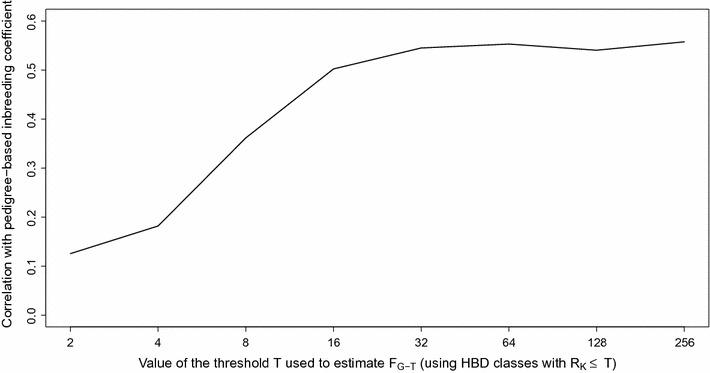
Correlations between the inbreeding coefficients estimated with respect to different base populations (*F*_*G*-*T*_) and the inbreeding coefficient estimated from pedigree data for the Belgian Blue sires born after 1999 and using the HD panel. Different base populations were obtained by selecting different thresholds *T* that determine which HBD-classes are considered for estimating *F*_*G*-*T*_ (e.g., setting the base population approximately 0.5 * *T* generations in the past). The corresponding inbreeding coefficients *F*_*G*-*T*_ are estimated as the probability of belonging to any of the HBD classes with a *R*_*k*_ ≤ *T* averaged over the whole genome. Genomic inbreeding coefficients were estimated with the Mix14R model

Correlations of these estimates with pedigree inbreeding coefficients (considering only individuals born after 1999 to increase pedigree depth) are also in Table 5. Overall correlations were moderate with the highest values for correlations with homozygosity and ROH-based measures (0.55 for both measures) and slightly lower values for those with the HMM-based estimator (0.46), whereas we observed a low relationship with F_GRM_ (0.29) and a moderate correlation with F_UNI_ (0.45). We also compared the F_PED_ and inbreeding coefficients estimated with our model with respect to different base populations (Fig. 8) and found that correlations increased up to F_G-32_ (capturing the inbreeding from ancestors approximately 16 generations back) and then presented a plateau from F_G-32_ to F_G-256_ reaching a maximum at r = 0.56 (i.e., slightly better than homozygosity-based estimators). This trend was expected since F_PED_ is estimated for a limited number of generations back in time. The average equivalent number of known generations estimated with PEDIG [43] was 6.3 for the bulls born after 1999 (it increased from 5.5 for bulls born in 2000 to 7.5 for those born in 2010) corresponding on average to F_G-16_. The addition of HBD-class *R*_*k*_ = 32 allows the capture of contributions from some older branches of the pedigree and the smallest HBD segments inherited from common ancestors in the pedigree.

## Discussion

For several reasons, the Belgian Blue Beef cattle breed is considered as an extremely selected breed. It is famous for its exceptional muscular development referred to as “double muscling”, which is caused by an 11-bp deletion in the *myostatin* gene [44]. This loss-of-function variant is almost fixed in the current population (e.g., [45]) but muscular development was further improved through intense selection [46]. As a result, most often calving requires caesarian section. In addition, artificial insemination is more frequent in this breed compared to other beef cattle breeds, which allows a more intense use of key sires. In recent years, several outbursts of recessive defects associated with inbreeding have been reported. For instance, causative variants were identified for eight recessive defects including congenital muscular dystonia 1 and 2 [5], crooked tail syndrome [6, 7], stunted growth [8], gingival hamartome [9], prolonged gestation, lethal arthrogryposis syndrome [10] and junctional epidermolysis bullosa [11]. Some of these defects have reached a high frequency in the population.

When estimated over all HBD-classes, the average genomic inbreeding coefficient was high (higher than 0.30) but these values were comparable to those obtained for other cattle breeds of European origin (i.e., BBB presented intermediate values). In agreement with Purfield et al. [22], samples from breeds that originated from the British Isles (Hereford, Angus, Jersey or Guernsey) presented high inbreeding coefficients (≈ 34 to 40%), possibly as a result of closed population histories and strict importation restrictions [22]. Similarly, high levels of inbreeding in Holstein and Brown Swiss breeds were previously reported [21, 41, 47]. When focusing on recent common ancestors only (associated with HBD-classes with *R*_*k*_ ≤ 64), we observed lower inbreeding coefficients in BBB cattle, ranging from 1.0 to 17.7% across animals (6.8% on average), with a positive trend: animals from the current population presenting 6% higher inbreeding coefficients on average than individuals born 30 years ago. Some individuals accumulated more than 10% recent autozygosity and carried HBD segments longer than 10 or even 50 Mb. The same model applied to other species, i.e., dog breeds or sheep populations that suffered severe bottlenecks revealed significantly higher levels of recent autozygosity [26]. Conversely, some human and sheep populations presented lower levels of recent autozygosity (even lower than 1% on average). The recent HBD-classes are probably more relevant for management purposes because they account for most of the individual variation in genome-wide autozygosity. In addition, deleterious variants might be mostly associated to recent HBD segments because older variants have undergone more generations of selection against deleterious effects (e.g., [3, 48, 49]). Recent intensive selection of key sires allowed some deleterious variants to reach higher frequency than under natural selection. Indeed, strong bottlenecks that occur with domestication, breed creation or intensive selection in cattle result in the relaxation of purifying selection and increase the load of deleterious mutations (e.g., [50, 51]). For instance, all identified variants that cause recessive defects in BBB cattle are specific to this breed (suggesting their young age). We applied our model to previously genotyped cases (see [52]) and the causative variants were found on recent HBD segments (associated with HBD-classes with *R*_*k*_ ≤ 32), also suggesting that these variants are relatively young.

Application of our model with different SNP densities showed large differences in average estimated inbreeding coefficients, with the average F_G_ equal to 0.060, 0.093 and 0.303 using the LD, 50 K and HD panel, respectively. Correlations between these estimates were very high (even with the LD panel, r > 0.93). High-density panels allow the capture of shorter ROH that are associated with very ancient ancestors, are characteristic of the population (associated with past demographic history) and present little individual variation. For recent HBD classes, estimators were similar across SNP panels (up to *R*_*k*_ = 32 with the LD panel and 256 with the 50 K panel). Small HBD segments, ranging from 10 kb to 1 Mb, accounted for most of the differences obtained with the HD panel compared to the lower density panels. A substantial proportion of HBD segments longer than respectively 1 and 5 Mb were identified with the LD and the 50 K panels. These observations are consistent with those of Ferenčaković et al. [20] and Purfield et al. [22] who showed that denser panels can be used to identify short ROH and that the 50 K panel proved suitable to identify ROH longer than 5 Mb. If the goal is to estimate the inbreeding coefficient with respect to a recent base population, which has more variation and is possibly the most functionally relevant one (see above), these LD and 50 K panels provide enough information (e.g., the correlation between F_G_ estimated with the HD and the 50 K panels was equal to 0.975). Regarding the optimal model, our comparisons indicated that models with a few HBD classes (1 or 2 according to SNP density) achieved results that were as good as those obtained with 13 HBD classes in terms of F_G_ and correlations with more complex models. Thus, such parsimonious models were selected based on the BIC. For each SNP panel, we recommend the use of the largest *K* that is optimal for a substantial proportion of individuals since that value is required for these animals and using a larger *K* will not penalize the other individuals. To make comparisons between individuals easier, we also recommend the use of a model with pre-defined *R*_*k*_ rates and the same HBD-classes for all individuals. In that case, the use of at least two HBD-classes is preferable with low-density panels, one to capture the recent HBD segments and one that is associated with more remote ancestors. Three HBD-classes models present a parsimonious solution to distinguish recent from ancient autozygosity (similarly to [16]) but if the objective is to obtain a finer age-based partitioning of autozygosity, more HBD classes could be recommended.

Comparisons of inbreeding coefficients obtained with different estimators have already been reported in the literature. In this paper, we also report correlations with our estimates of genome-wide inbreeding. These comparisons are essentially indicative since different methods refer to different base populations and all estimators are not fully comparable (e.g., [53]). In addition, some estimators are sensitive to the estimated allelic frequencies. Here, we used frequencies that were estimated using the set of genotyped bulls born before 1985. At moderate to high SNP density, the genome-wide inbreeding coefficient estimated with our model, averaged over all SNPs and HBD classes, was highly correlated with homozygosity measures or ROH-based estimates, whereas lower correlations were obtained when compared to estimates based on the genomic relationship matrix. Low correlations between F_GRM_ and homozygosity measures (homozygosity or ROH) were previously reported (e.g., [54]) although moderate to high correlations were also found (e.g., [2, 4]). It should be kept in mind that these results must be interpreted with caution because global estimators, and particularly F_GRM_, depend strongly on the estimation of allele frequencies in the population. In addition to global inbreeding coefficients, our model also estimates local autozygosity (i.e., it identifies HBD segments) and uses the linkage between SNPs as ROH-based estimators, conversely to global estimators that consider all SNPs as independent (F_HOM_, F_GRM_, F_UNI_ or F_PED_). Correlations with homozygosity measures decreased at lower SNP densities when the use of linkage between successive SNP positions was more important to determine whether a position is IBD or not. ROH-based estimators are not frequently used with LD panels in cattle and previous studies concluded that LD panels were appropriate to identify recent inbreeding or HBD segments longer than 5 Mb [20, 22]. The HMM proved to work well with LD panels, i.e., it captured the recent HBD segments, presented high correlations with coefficients estimated at higher density, and provided HBD probabilities. It is indeed recommended to use such probabilities at low-density because they account for uncertainty due to lower informativeness as opposed to ROH-based classification or the Viterbi algorithm. We showed that, at lower SNP densities, the smallest HBD segments are not captured but also that the Viterbi algorithm even fails to identify some segments of moderate size. Therefore, we recommend the use of HBD probabilities that are obtained with the forward-backward algorithm. Most of the global estimators provided inbreeding coefficients relative to a base population, i.e., the founders of the pedigree or the population used to estimate allele frequencies, whereas the multiple-HBD class model provides an age-based partitioning of autozygosity. As a result, inbreeding coefficients estimated by including all HBD classes are higher because some HBD-classes trace back to more remote generations than the base population commonly used by other methods and the SNP density determines how ancient HBD segments can be captured. Compared to rule-based ROH, the HMM framework also allows to accommodate low-fold sequencing or genotype-by-sequencing data, i.e., when genotypes are not unambiguously determined, as described in Vieira et al. [25] and Druet and Gautier [26].

Moderate to high correlations between F_PED_ and F_ROH_ (from 0.50 to 0.75) were reported in cattle (e.g., [3, 21, 22, 54, 55]). In addition, long ROH (> 5 Mb) were shown to be closely associated with pedigree inbreeding coefficients [22]. Correlations between estimators obtained from the pedigree and the genomic relationship matrices are more variable, ranging from moderate (e.g., [4]) to high (e.g., [37]), whereas in other studies, these correlations were particularly low [54, 56]. As mentioned above, these differences might be due to the estimation of the allelic frequencies. Inbreeding coefficients estimated with the HMM had moderate correlations with pedigree-based inbreeding coefficients, lower than with methods based on homozygosity or ROH that were in the range with correlations reported in previous studies. However, correlations were higher with the autozygosity associated to the recent HBD-classes, which is a desired feature since these recent classes correspond to the autozygosity captured by the pedigree whereas old HBD-classes are associated to ancestors tracing further back than the genealogy. Similarly, correlations between HMM inbreeding coefficients estimated with the LD panel and pedigree-based estimates were higher since they capture only recent autozygosity (compared to higher density panels).

## Conclusions

Although we observed high levels of inbreeding associated with small HBD segments in Belgian Blue Beef cattle, recent HBD segments account for most of the individual variation. Recent autozygosity can be captured efficiently with low-density SNP panels and with relatively simple models (e.g., two HBD classes) although we recommend the use of models with pre-defined *R*_*k*_ rates that are associated with the expected length of HBD segments (the same HBD-classes are then used for all individuals) to make comparisons between individuals easier. More complex models (with more age-based HBD classes) are needed to obtain a finer age-based partitioning of inbreeding levels and indications of the past demographic history of a population. Such partitioning allows to better understand which HBD classes contribute to individual autozygosity. In addition, the use of more classes avoids the fragmentation of long HBD segments into smaller fragments with next-generation sequencing data. Estimates obtained with the HMM framework are highly correlated with those obtained based on relative homozygosity (or ROH). In addition, such HMM can use genotype probabilities (e.g., with low-fold sequencing data) and provide, beyond global estimates, local HBD probabilities that are still useful at lower SNP densities. Such local HBD probabilities might be useful to identify regions associated with inbreeding depression.

## Additional files

**Additional file 1. Figure S1.** Trend per year of birth of individual inbreeding coefficients in the 634 Belgian Blue sires. Inbreeding coefficients were estimated with the Mix14R model (13 HBD-classes model with pre-defined *R*_*k*_ rates) using the BovineHD genotyping panel. (a) Trend for genomic inbreeding coefficients estimated using all HBD classes; (b) trend for genomic inbreeding coefficients estimated with the most recent HBD classes (*R*_*k*_ ≤ 32) and (c) trend obtained with pedigree-based estimates.

**Additional file 2.** Boxplots of proportions of individual genomes associated with 13 HBD-classes with pre-defined *R*_*k*_ rates (MIX14R model) in 11 cattle breeds of European origin using the BovineHD genotyping panel. The proportions correspond to individual genome-wide probabilities of belonging to each of the HBD-classes.

**Additional file 3. Figure S2.** Comparison of genomic inbreeding coefficients estimated with different marker densities (LD panel in black, 50 K panel in red, BovineHD panel in green and WGS panel in blue) and for different base populations. Genomic inbreeding coefficients were estimated with the Mix14R model (13 HBD-classes model with pre-defined *R*_*k*_ rates) for 634 Belgian Blue sires. Different base populations were obtained by selecting different thresholds *T* that determine which HBD-classes were considered in the estimation of *F*_*G*-*T*_ (e.g., setting the base population approximately 0.5 * *T* generations in the past).

**Additional file 4. Figure S3.** Distribution of length of HBD segments identified with whole-genome sequence data and using models with different numbers of HBD classes.

**Additional file 5. Table S1.** Correlation coefficients between inbreeding coefficients estimated with different methods for the 634 Belgian Blue sires and using the 50 K panel. The table reports the correlations between all inbreeding coefficients estimated with different methods using the 50 K panel. **Table S2.** Correlation coefficients between inbreeding coefficients estimated with different methods for the 634 Belgian Blue sires and using the LD panel. The table reports the correlations between all inbreeding coefficients estimated with different methods using the LD panel.
