# Supplementary figures and images for: Age-based partitioning of individual genomic inbreeding levels in Belgian Blue cattle
Source: Genet Sel Evol. 2017 Dec 22;49:92. doi: 10.1186/s12711-017-0370-x (PMC5741860; doi:10.1186/s12711-017-0370-x)

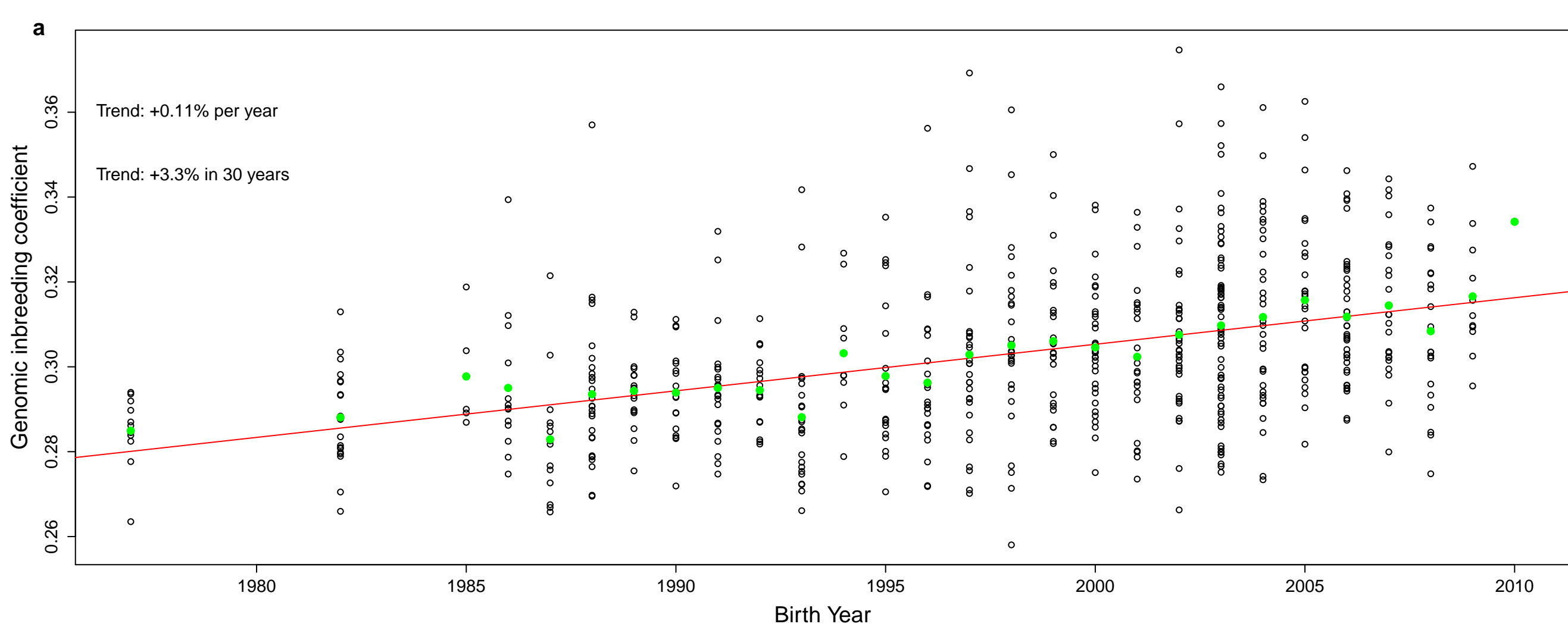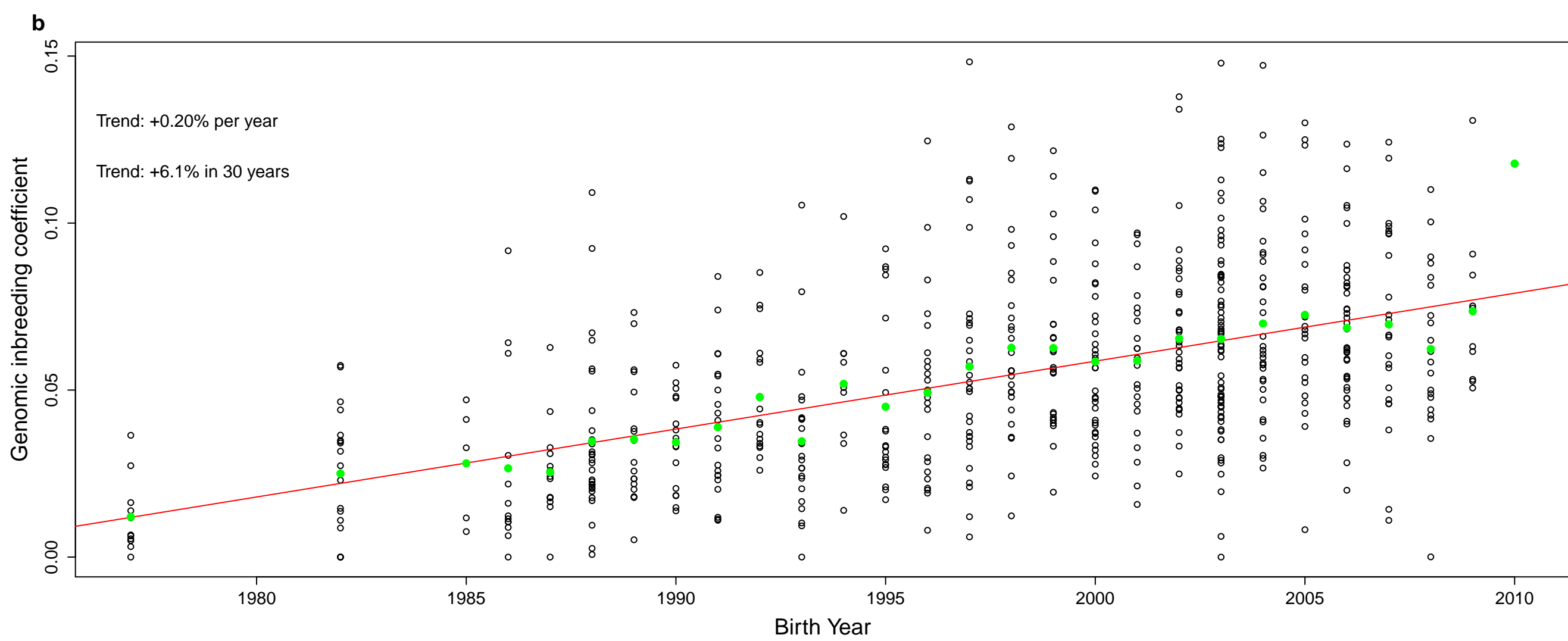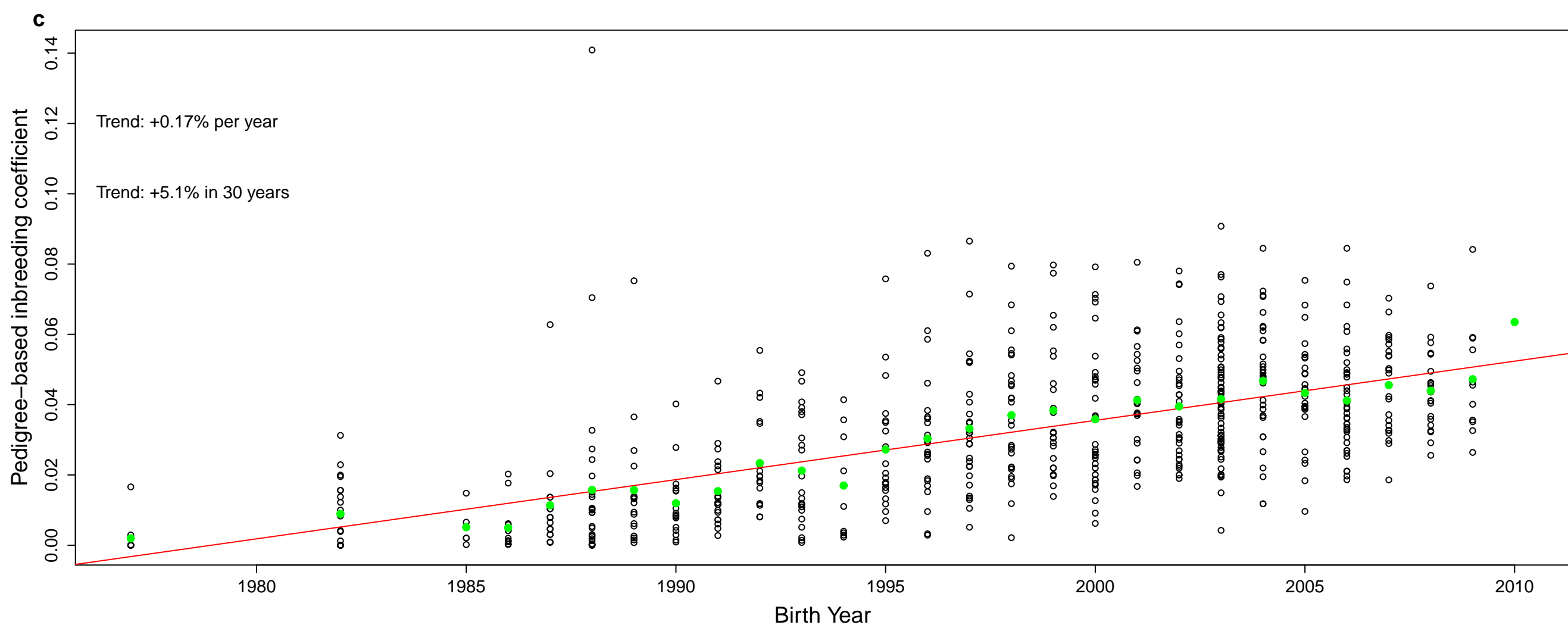

Supplement: Supplementary file 1 — Additional file 1. Figure S1. Trend per year of birth of individual inbreeding coefficients in the 634 Belgian Blue sires. Inbreeding coefficients were estimated with the Mix14R model (13 HBD-classes model with pre-defined R k rates) using the BovineHD genotyping panel. (a) Trend for genomic inbreeding coefficients estimated using all HBD classes; (b) trend for genomic inbreeding coefficients estimated with the most recent HBD classes (R k ≤ 32) and (c) trend obtained with pedigree-based estimates. [file 12711_2017_370_MOESM1_ESM.pdf]

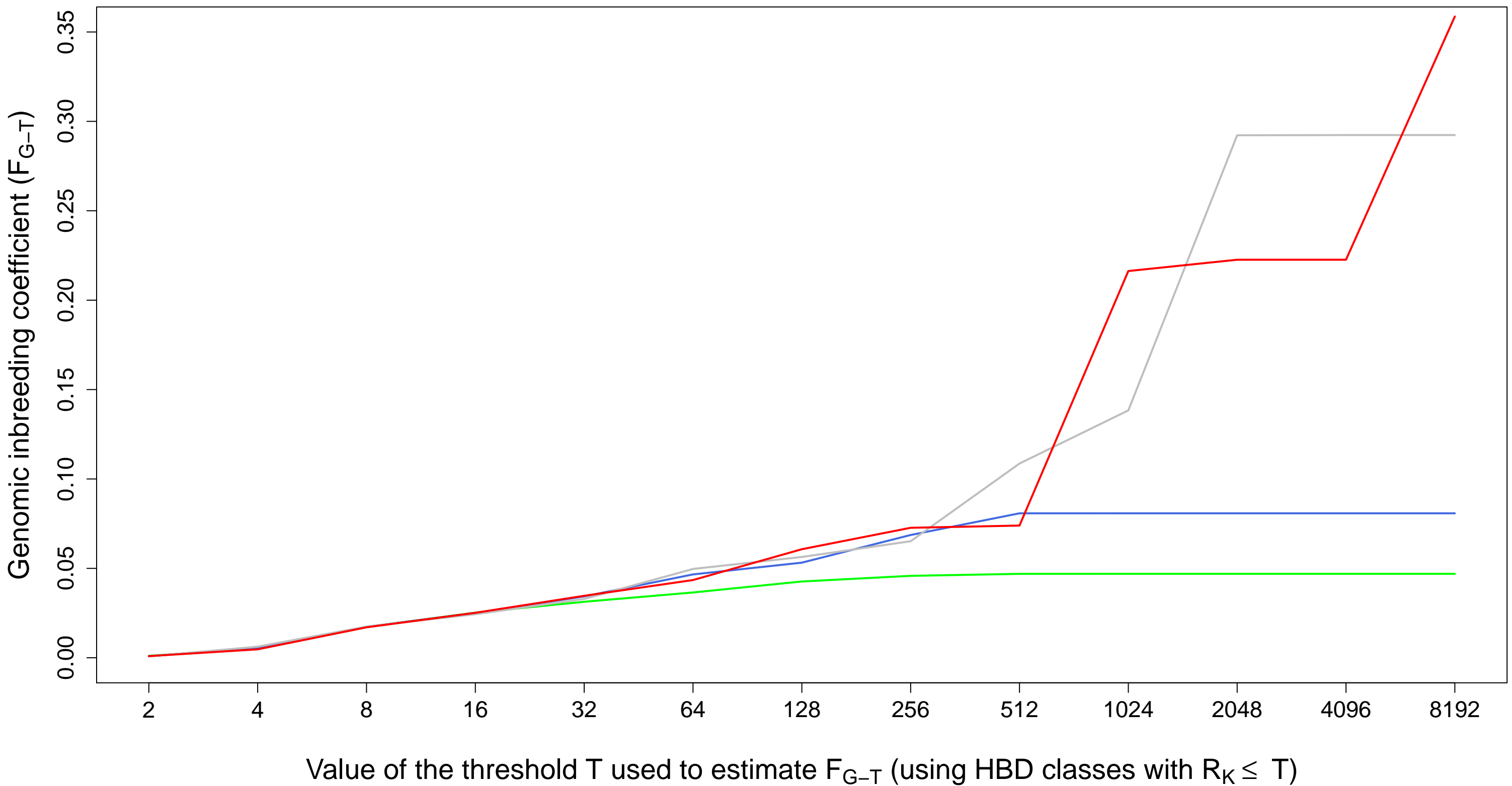

Supplement: Supplementary file 3 — Additional file 3. Figure S2. Comparison of genomic inbreeding coefficients estimated with different marker densities (LD panel in black, 50 K panel in red, BovineHD panel in green and WGS panel in blue) and for different base populations. Genomic inbreeding coefficients were estimated with the Mix14R model (13 HBD-classes model with pre-defined R k rates) for 634 Belgian Blue sires. Different base populations were obtained by selecting different thresholds T that determine which HBD-classes were considered in the estimation of F G-T (e.g., setting the base population approximately 0.5 * T generations in the past). [file 12711_2017_370_MOESM3_ESM.pdf]

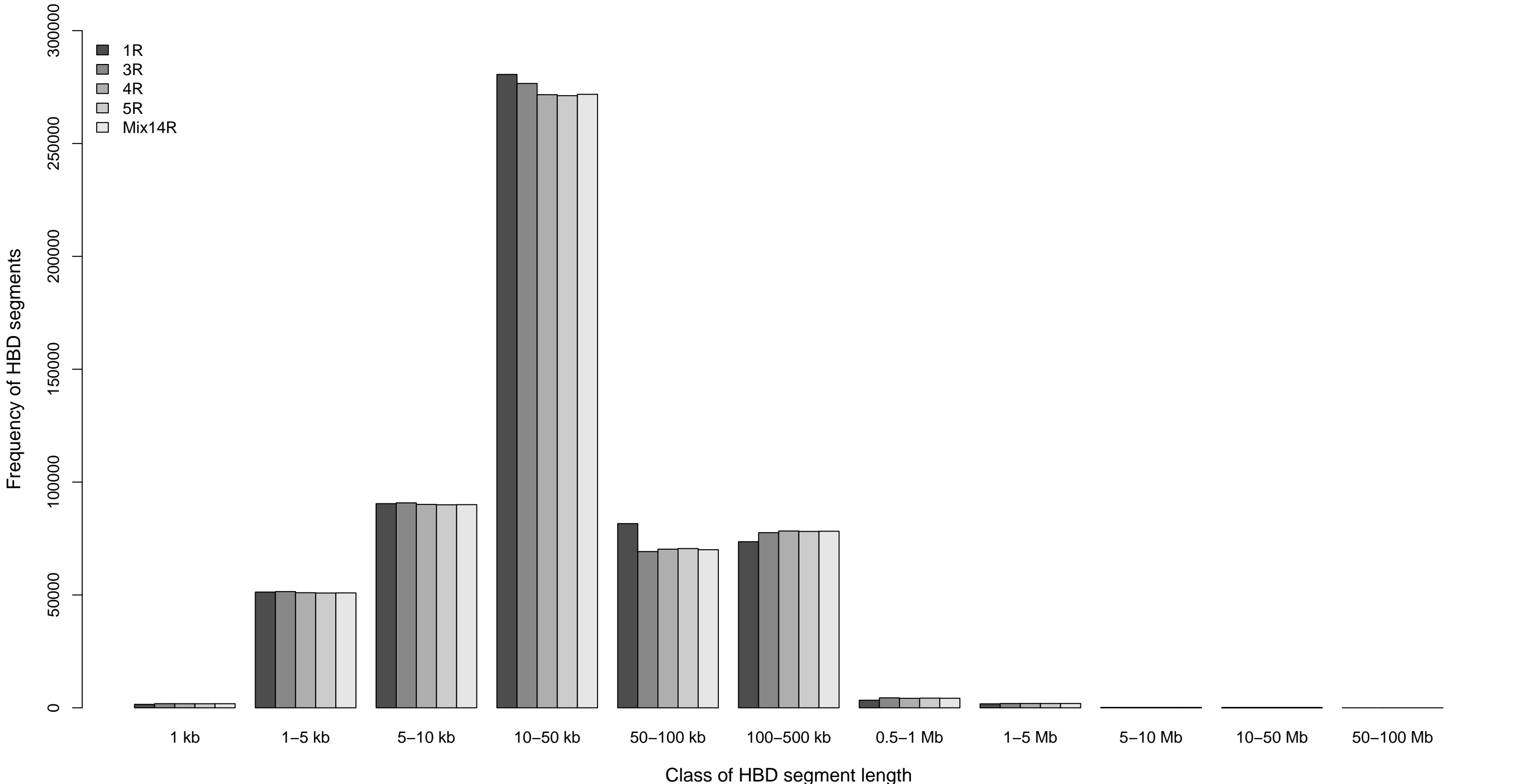

Supplement: Supplementary file 4 — Additional file 4. Figure S3. Distribution of length of HBD segments identified with whole-genome sequence data and using models with different numbers of HBD classes. [file 12711_2017_370_MOESM4_ESM.pdf]
