# Supplementary material for: Age-based partitioning of individual genomic inbreeding levels in Belgian Blue cattle
Source: Genet Sel Evol. 2017 Dec 22;49:92. doi: 10.1186/s12711-017-0370-x (PMC5741860; doi:10.1186/s12711-017-0370-x)
Supplement: Supplementary file 2 — Additional file 2. Boxplots of proportions of individual genomes associated with 13 HBD-classes with pre-defined R k rates (MIX14R model) in 11 cattle breeds of European origin using the BovineHD genotyping panel. The proportions correspond to individual genome-wide probabilities of belonging to each of the HBD-classes. [file 12711_2017_370_MOESM2_ESM.pdf]

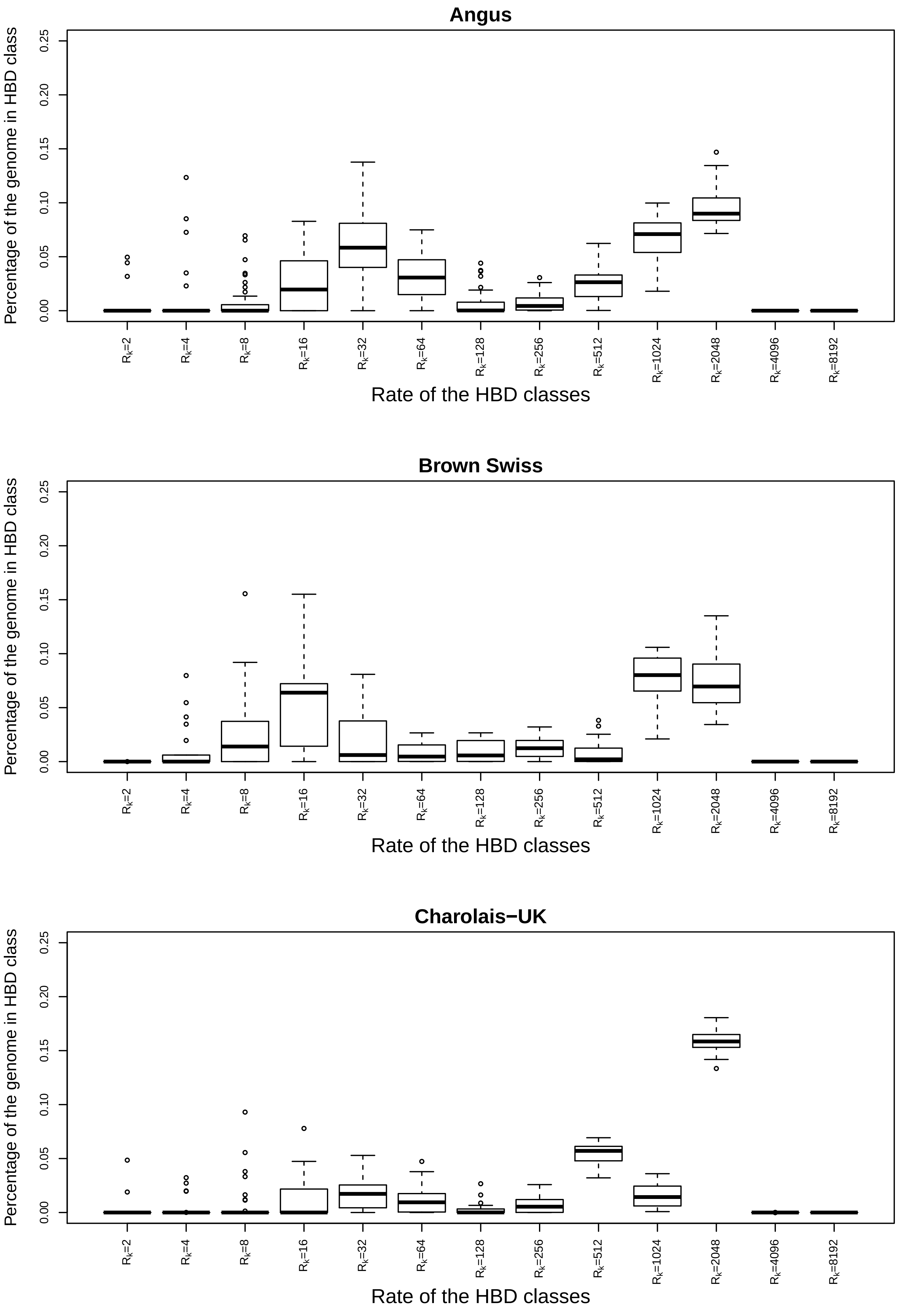

Angus
Brown Swiss
Charolais−UK
Percentage of the genome in HBD class
Rate of the HBD classes
0.00
0.05
0.10
0.15
0.20
0.25
Rk=2
Rk=4
Rk=8
Rk=16
Rk=32
Rk=64
Rk=128
Rk=256
Rk=512
Rk=1024
Rk=2048
Rk=4096
Rk=8192

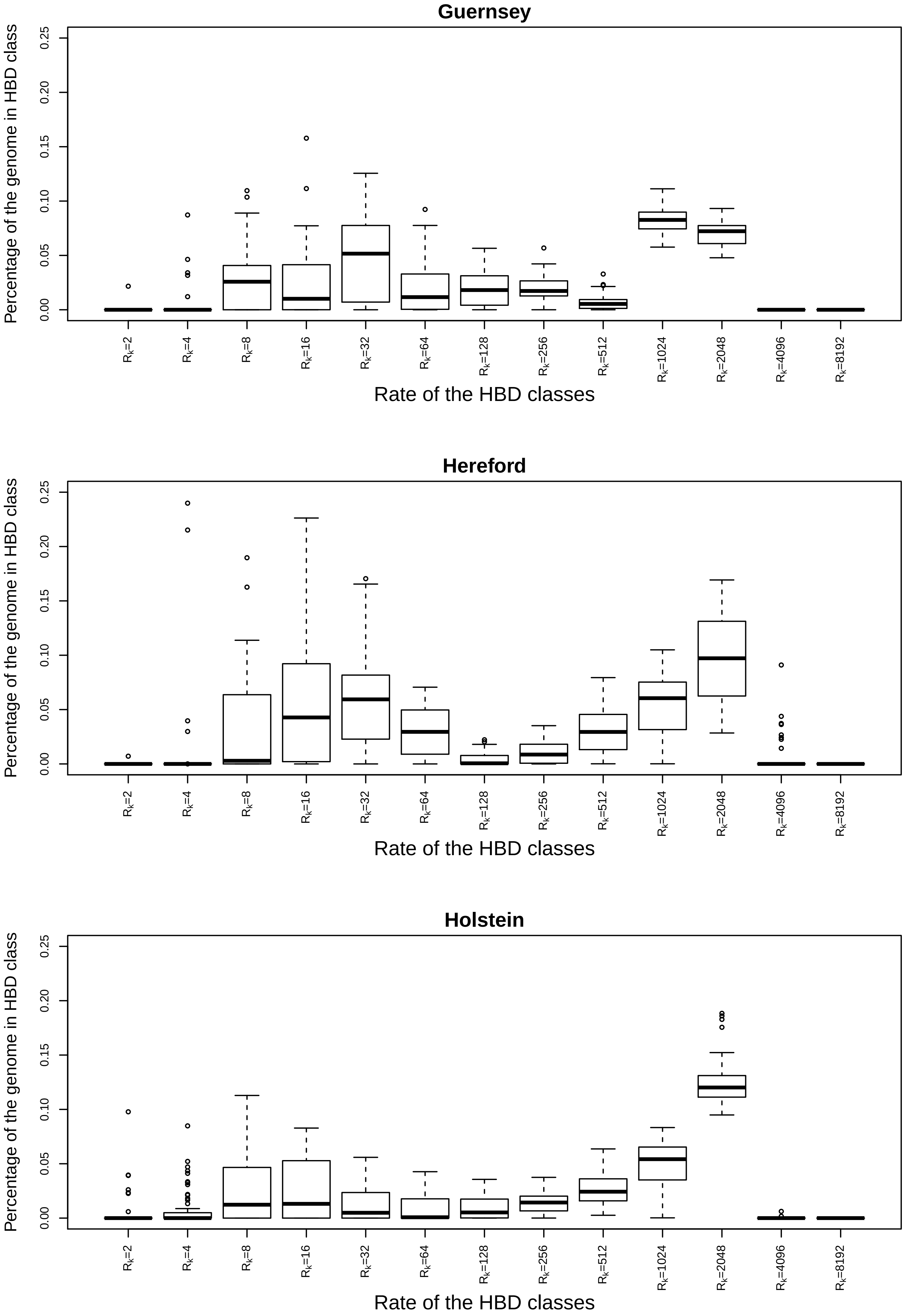
Guernsey
Percentage of the genome in HBD class
0.00
0.05
0.10
0.15
0.20
0.25
R_k=2
R_k=4
R_k=8
R_k=16
R_k=32
R_k=64
R_k=128
R_k=256
R_k=512
R_k=1024
R_k=2048
R_k=4096
R_k=8192
Rate of the HBD classes
Hereford
Percentage of the genome in HBD class
0.00
0.05
0.10
0.15
0.20
0.25
R_k=2
R_k=4
R_k=8
R_k=16
R_k=32
R_k=64
R_k=128
R_k=256
R_k=512
R_k=1024
R_k=2048
R_k=4096
R_k=8192
Rate of the HBD classes
Holstein
Percentage of the genome in HBD class
0.00
0.05
0.10
0.15
0.20
0.25
R_k=2
R_k=4
R_k=8
R_k=16
R_k=32
R_k=64
R_k=128
R_k=256
R_k=512
R_k=1024
R_k=2048
R_k=4096
R_k=8192
Rate of the HBD classes

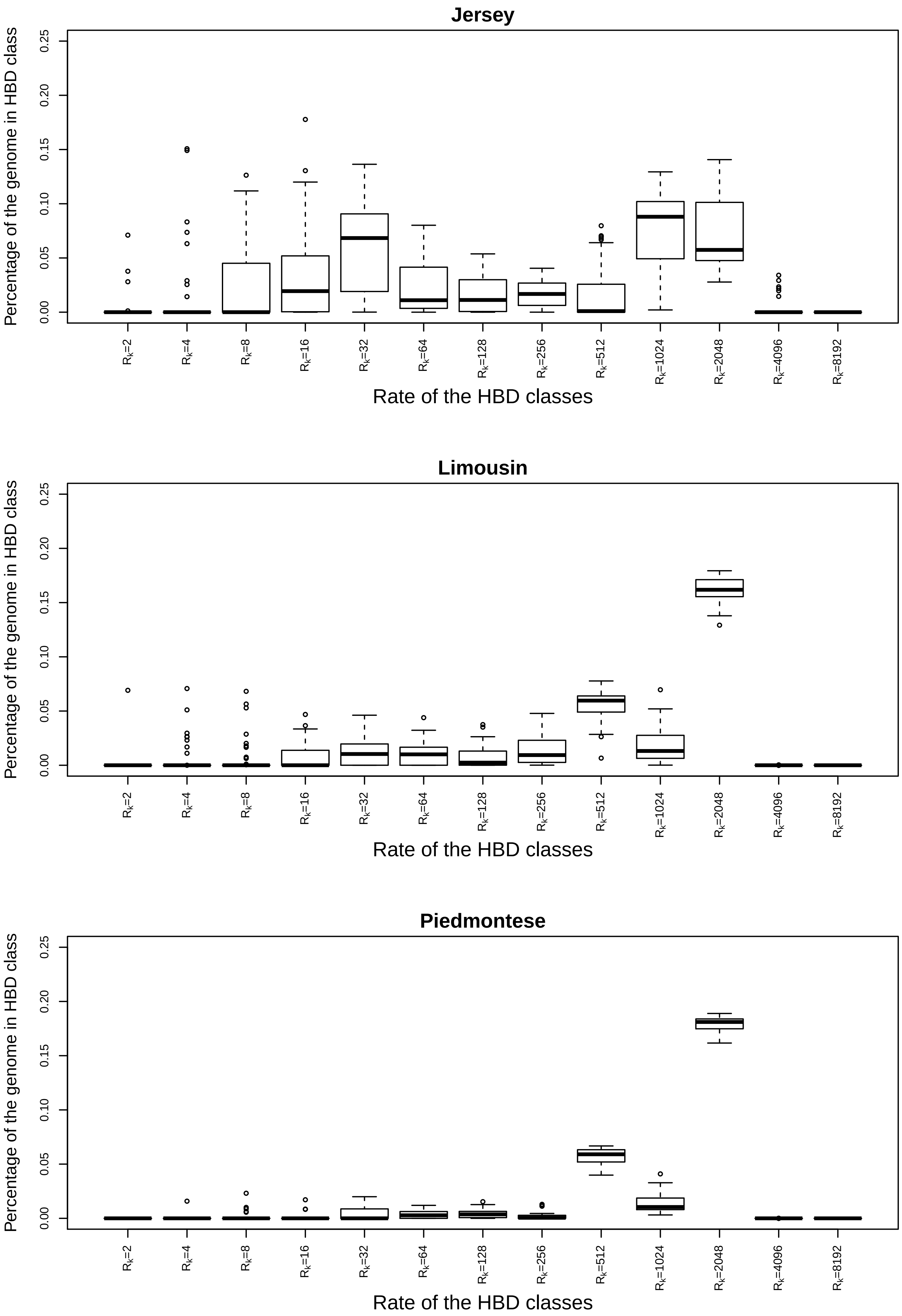
Jersey
Limousin
Piedmontese
Percentage of the genome in HBD class
Rate of the HBD classes
0.00
0.05
0.10
0.15
0.20
0.25
$R_k$=2
$R_k$=4
$R_k$=8
$R_k$=16
$R_k$=32
$R_k$=64
$R_k$=128
$R_k$=256
$R_k$=512
$R_k$=1024
$R_k$=2048
$R_k$=4096
$R_k$=8192

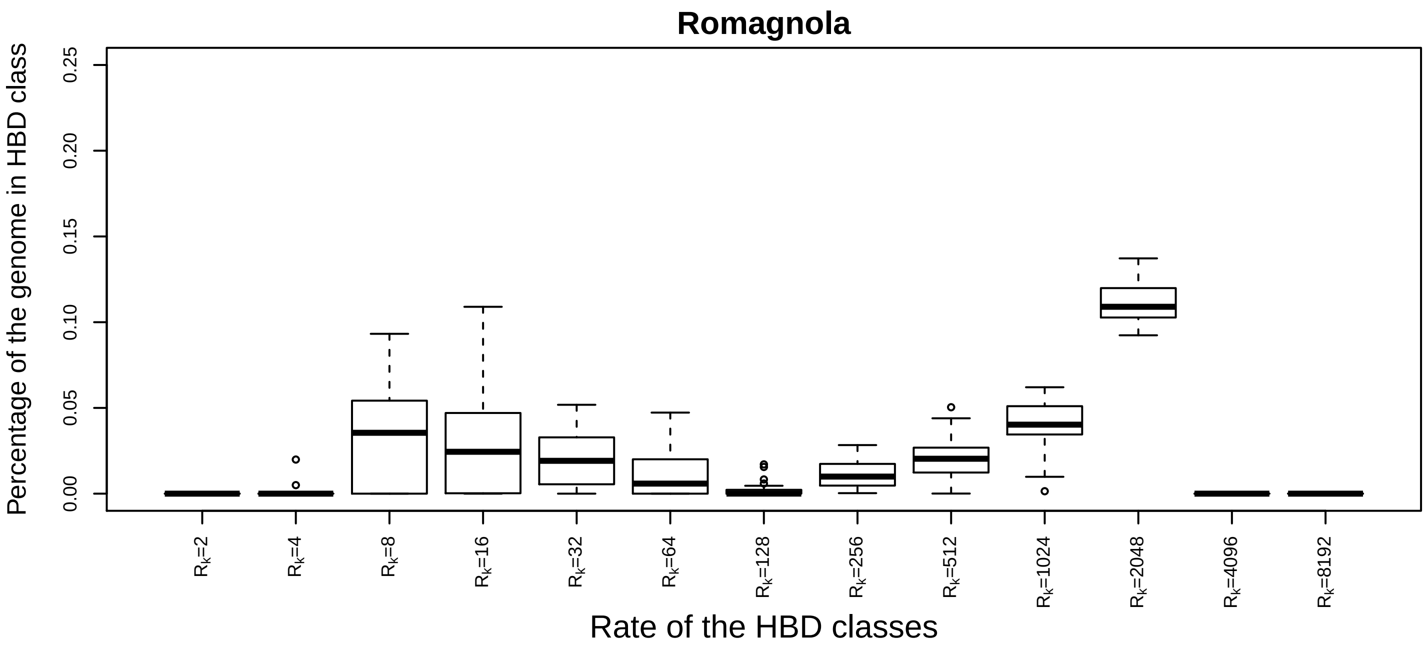

Romagnola
Percentage of the genome in HBD class
0.00
0.05
0.10
0.15
0.20
0.25
R_k=2
R_k=4
R_k=8
R_k=16
R_k=32
R_k=64
R_k=128
R_k=256
R_k=512
R_k=1024
R_k=2048
R_k=4096
R_k=8192
Rate of the HBD classes
