# Supplementary material for: Age-based partitioning of individual genomic inbreeding levels in Belgian Blue cattle
Source: Genet Sel Evol. 2017 Dec 22;49:92. doi: 10.1186/s12711-017-0370-x (PMC5741860; doi:10.1186/s12711-017-0370-x)
Supplement: Supplementary file 5 — Additional file 5. Table S1. Correlation coefficients between inbreeding coefficients estimated with different methods for the 634 Belgian Blue sires and using the 50 K panel. The table reports the correlations between all inbreeding coefficients estimated with different methods using the 50 K panel. Table S2. Correlation coefficients between inbreeding coefficients estimated with different methods for the 634 Belgian Blue sires and using the LD panel. The table reports the correlations between all inbreeding coefficients estimated with different methods using the LD panel. [file 12711_2017_370_MOESM5_ESM.docx]

Table S1. Correlation coefficients between individual inbreeding coefficients estimated with different methods for the 634 Belgian Blue sires and using the 50K panel. The table reports the correlations between all inbreeding coefficients estimated with different methods using the 50K panel.

|  | **F_HOM_** | **F_ExHOM_** | **F_ROH_** | **F_GRM1_** | **F_GRM2_** | **F_UNI_** | **F_PED_** |
| --- | --- | --- | --- | --- | --- | --- | --- |
| **F_G_** | 0.921 | 0.917 | 0.925 | 0.736 | 0.447 | 0.890 | 0.463 |
| **F_HOM_** |  | 0.999 | 0.940 | 0.633 | 0.307 | 0.875 | 0.517 |
| **F_ExHOM_** |  |  | 0.941 | 0.639 | 0.315 | 0.880 | 0.518 |
| **F_ROH_** |  |  |  | 0.613 | 0.296 | 0.837 | 0.527 |
| **F_GRM1_** |  |  |  |  | 0.911 | 0.910 | 0.283 |
| **F_GRM2_** |  |  |  |  |  | 0.710 | 0.075 |
| **F_UNI_** |  |  |  |  |  |  | 0.452 |

F_G_ = inbreeding coefficient estimated as the probability to belong to any of the HBD classes averaged over the whole genome; F_HOM_ = inbreeding coefficient based on the proportion of homozygous SNPs; F_ExHOM_ = excess homozygosity estimator; F_ROH_ = inbreeding coefficient estimated as proportion of the genome captured by ROH; F_GRM1_ = inbreeding coefficient based on the diagonal elements of genomic relationship matrix (dividing all SNP contribution by the same denominator); F_GRM2_ = inbreeding coefficient based on the diagonal elements of genomic relationship matrix (dividing each SNP by its own weight 2f_i_(1-f_i_), f_i_ being the allele frequency for allele i); F_UNI_ = inbreeding coefficient based on the correlation between uniting gametes; F_PED_ = inbreeding coefficient estimated from pedigree data.

Table S2. Correlation coefficients between individual inbreeding coefficients estimated with different methods for the 634 Belgian Blue sires and using the LD panel. The table reports the correlations between all inbreeding coefficients estimated with different methods using the LD panel.

|  | **F_HOM_** | **F_ExHOM_** | **F_ROH_** | **F_GRM1_** | **F_GRM2_** | **F_UNI_** | **F_PED_** |
| --- | --- | --- | --- | --- | --- | --- | --- |
| **F_G_** | 0.863 | 0.862 | 0.789 | 0.673 | 0.550 | 0.845 | 0.518 |
| **F_HOM_** |  | 1.000 | 0.725 | 0.611 | 0.448 | 0.870 | 0.509 |
| **F_ExHOM_** |  |  | 0.726 | 0.612 | 0.450 | 0.871 | 0.511 |
| **F_ROH_** |  |  |  | 0.501 | 0.401 | 0.668 | 0.396 |
| **F_GRM1_** |  |  |  |  | 0.962 | 0.908 | 0.275 |
| **F_GRM2_** |  |  |  |  |  | 0.817 | 0.146 |
| **F_UNI_** |  |  |  |  |  |  | 0.420 |

F_G_ = inbreeding coefficient estimated as the probability to belong to any of the HBD classes averaged over the whole genome; F_HOM_ = inbreeding coefficient based on the proportion of homozygous SNPs; F_ExHOM_ = excess homozygosity estimator; F_ROH_ = inbreeding coefficient estimated as proportion of the genome captured by ROH; F_GRM1_ = inbreeding coefficient based on the diagonal elements of genomic relationship matrix (dividing all SNP contribution by the same denominator); F_GRM2_ = inbreeding coefficient based on the diagonal elements of genomic relationship matrix (dividing each SNP by its own weight 2f_i_(1-f_i_), f_i_ being the allele frequency for allele i); F_UNI_ = inbreeding coefficient based on the correlation between uniting gametes; F_PED_ = inbreeding coefficient estimated from pedigree data.
